# Cross-seeding between the functional amyloidogenic CRES and CRES3 family members and their regulation of Aβ assembly

**DOI:** 10.1074/jbc.RA120.015307

**Published:** 2021-01-09

**Authors:** Hoa Quynh Do, Aveline Hewetson, Collin G. Borcik, Mary Catherine Hastert, Sandra Whelly, Benjamin J. Wylie, Roger Bryan Sutton, Gail A. Cornwall

**Affiliations:** 1 Department of Cell Biology and Biochemistry, Texas Tech University Health Sciences Center, Lubbock, Texas, USA; 2 Department of Chemistry and Biochemistry, Texas Tech University, Lubbock, Texas, USA; 3 College of Arts and Sciences Microscopy, Texas Tech University, Lubbock, Texas, USA; 4 Department of Cell Physiology and Molecular Biophysics, Texas Tech University Health Sciences Center, Lubbock, Texas, USA

**Keywords:** cystatin, CRES subgroup, amyloid, epididymis, cross-seeding, mouse, Aβ, CRES, cystatin-related epididymal spermatogenic, DMSO, dimethyl sulfoxide, FTIR, Fourier transform infrared spectroscopy, MES, (2-(4-morpholino)-ethane sulfonic acid), PAD, protein aggregation disease, PVDF, polyvinylidene fluoride, RIP1, RIP3, Receptor-interacting serine/threonine-protein kinase 1, 3, SSNMR, solid-state nuclear magnetic resonance, TEM, transmission electron microscopy, TIDE, T2∗-weIghted DEconvolution, ZP, zona pellucida

## Abstract

Accumulating evidence shows that amyloids perform biological roles. We previously showed that an amyloid matrix composed of four members of the CRES subgroup of reproductive family 2 cystatins is a normal component of the mouse epididymal lumen. The cellular mechanisms that control the assembly of these and other functional amyloid structures, however, remain unclear. We speculated that cross-seeding between CRES members could be a mechanism to control the assembly of the endogenous functional amyloid. Herein we used thioflavin T assays and negative stain transmission electron microscopy to explore this possibility. We show that CRES3 rapidly formed large networks of beaded chains that possessed the characteristic cross-β reflections of amyloid when examined by X-ray diffraction. The beaded amyloids accelerated the amyloidogenesis of CRES, a less amyloidogenic family member, in seeding assays during which beads transitioned into films and fibrils. Similarly, CRES seeds expedited CRES3 amyloidogenesis, although less efficiently than the CRES3 seeding of CRES. These studies suggest that CRES and CRES3 hetero-oligomerize and that CRES3 beaded amyloids may function as stable preassembled seeds. The CRES3 beaded amyloids also facilitated assembly of the unrelated amyloidogenic precursor Aβ by providing a surface for polymerization though, intriguingly, CRES3 (and CRES) monomer/early oligomer profoundly inhibited Aβ assembly. The cross-seeding between the CRES subgroup members is similar to that which occurs between bacterial curli proteins suggesting that it may be an evolutionarily conserved mechanism to control the assembly of some functional amyloids. Further, interactions between unrelated amyloidogenic precursors may also be a means to regulate functional amyloid assembly.

Amyloids, defined as proteins that assemble into highly ordered cross β-sheet structures, have historically been considered as pathological assemblies that play causative roles in neurodegenerative diseases and prionopathies. Amyloids, however, also carry out biological roles in species ranging from bacteria to human (1, 2). These functional amyloids serve as biological scaffolds, signaling complexes, and storage depots and include functions in host defense, germline specification, long-term memory, melanin synthesis, sperm clearance, and fertilization (3, 4, 5, 6, 7, 8, 9). Because functional and pathological amyloids follow similar aggregation pathways, it is unclear how functional amyloids polymerize into their mature structures *in vivo* and avoid the cytotoxicity that can be associated with the intermediate oligomeric amyloid forms. It is generally thought, however, that functional amyloids form under controlled conditions while pathological amyloid assembly is uncontrolled (10).

Several mechanisms have been proposed that would allow a cell to regulate when and where a biological amyloid forms while minimizing its exposure to possible cytotoxic conformers. These include confining amyloid formation to cellular organelles such as secretory granules, melanosomes, and the sperm acrosome (3, 5, 6), rapid assembly kinetics (11, 12), activation by protein processing (13, 14), and/or by coordinated interactions between amyloidogenic family members with one acting as a nucleator to facilitate amyloidogenesis in the others (10, 15). Since many amyloidogenic precursors exhibit nucleation-dependent assembly, the presence of a preassembled seed could be a means to accelerate this process. Indeed, *Escherichia coli* and other Gram-negative bacteria require interactions between several amyloidogenic curli (csg) family members encoded by the csgBAC operon in order to form a functional extracellular amyloid matrix that participates in biofilm formation (16). Specifically, CsgB serves as a nucleator to accelerate the polymerization of monomeric CsgA in a highly regulated assembly process at the cell surface (17, 18). Cross-seeding between family members as a possible means to regulate functional amyloid assembly has not yet been established in mammals. However, recent studies showed that alternative splicing within the amyloidogenic repeat domain of human PMEL, which forms an amyloid integral for melanin synthesis, resulted in an isoform that served as a nucleator to facilitate the amyloidogenesis of the longer PMEL form (19). These studies revealed a different mechanism to achieve the same result of a controlled nucleated polymerization as in *E. coli*.

We previously showed that as spermatozoa migrate through the mouse epididymal tubule, they are surrounded by a complex extracellular amyloid matrix that likely participates in their maturation into functional cells as well as provides protection from pathogens that can ascend the male reproductive tract (20). This functional amyloid matrix is composed of multiple members of family 2 cystatins of cysteine protease inhibitors including cystatin C and four members (CRES, CRES2, CRES3, cystatin E2) of the CRES (cystatin-related epididymal spermatogenic) reproductive subgroup of cystatins (11, 21). All four subgroup members colocalize within the epididymal amyloid matrix suggesting they may hetero-oligomerize during matrix assembly (11). These results also imply that one or more CRES subgroup members might function as a seed to enable amyloidogenesis in other family members, possibly as a mechanism to regulate the building of the endogenous amyloid structure. Although CRES proteins exhibit only ∼20–30% sequence identity with each another, the AmylPred2 algorithm predicts all possess common amyloidogenic sites by which β-sheet assemblies could form (11, 22). Indeed, each subgroup member forms amyloid *in vitro* with its own distinct assembly properties. Of the four proteins, CRES is the least amyloidogenic while CRES3 is the most aggregation-prone and rapidly assembles *in vitro* into networks of large beaded chains that structurally resemble those formed by the prion domain of the Rnq1 yeast prion, a nucleator for other yeast prions (11, 23). Further, in the epididymis, CRES3 is associated only with the particulate fraction, while a proportion of CRES, CRES2, and cystatin E2 remains in the soluble fraction of the luminal fluid (11). These results suggest that *in vivo* CRES3 also has a greater propensity than other subgroup members to aggregate, and its presence only in the insoluble fraction may be functionally relevant.

Based on our findings, we hypothesized that CRES3 serves as a nucleator to facilitate amyloid assembly among other CRES subgroup members. Herein we performed cross-seeding experiments adding preformed CRES3 beaded chains to monomeric CRES and then following amyloid assembly by thioflavin T (ThT) fluorescence and transmission electron microscopy (TEM). We show that the CRES3 aggregates accelerated the assembly of CRES into higher-ordered amyloids in a dose-dependent manner that correlated with the loss of its beaded chains and the formation of new structures including films and fibrils. Similarly, CRES seeds templated the assembly of CRES3, although less robustly than the reverse experiment. Together our studies suggest that CRES3 and CRES hetero-oligomerize and that nucleated polymerization among family members may be an evolutionarily conserved mechanism to regulate the formation of some functional amyloids. In addition, we show that, depending on its structure, CRES3 regulated Aβ amyloidogenesis *via* both amyloid-promoting and amyloid-inhibiting functions. These studies imply that cross-seeding as well as inhibitory interactions can also occur between the CRES subgroup and unrelated amyloidogenic precursors, perhaps too as a mechanism to control the polymerization of functional structures.

## Results

### CRES3 forms SDS-stable and SDS-sensitive aggregates

**Figure 1:**
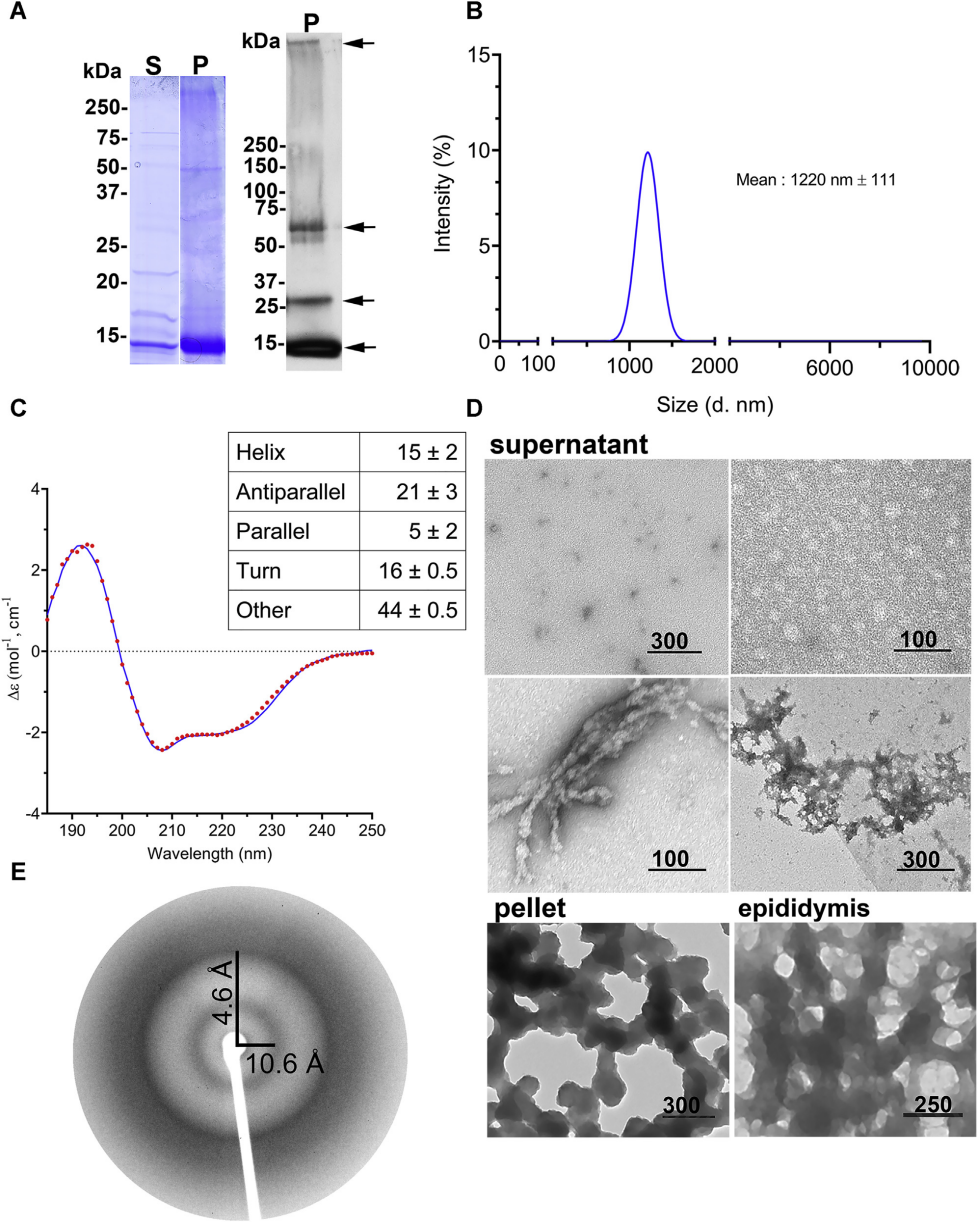
**CRES3 forms SDS-sensitive and SDS-resistant aggregates.***A*, Coomassie-stained SDS-PAGE of CRES3 supernatant (S) and pellet (P) (left panel) and western blot analysis of CRES3 pellet using an anti-CRES3 antibody (right panel). Following dilution into aqueous buffer and adjustment to neutral pH, CRES3 formed a white precipitate that was further separated into a soluble (supernatant) and insoluble (pellet) fraction by centrifugation. Arrows indicate CRES3 monomer and putative dimer, tetramer, and oligomers. *B*, dynamic light scattering of CRES3 soluble fraction showed particles >1000 nm diameter suggestive of oligomers. Data represent the mean ±SD diameter of particles from three independent CRES3 preparations. *C*, circular dichroism of CRES3 soluble fraction predicted a protein with mixed secondary structure containing antiparallel β-sheets. CD spectral curve shows experimental (*red dotted line*) and fitted data (*blue solid line*) from a representative CRES3 preparation. Table shows the mean ± SEM secondary structure as predicted from the spectral data by the BeStSel algorithm from n = 3 independent CRES3 preparations. *D*, negative stain TEM revealed a mixture of early amyloid assemblies in the CRES3 soluble/supernatant fraction while the insoluble/pellet contained only branched beaded chains. Data are representative of three independent CRES3 protein preparations. Similar branched beaded chains were detected by TEM in CD-1 mouse epididymal luminal fluid following binding and elution from the PAD ligand (epididymis). The beaded chain amyloids were not present in PAD pull-down of buffer only (data not shown). Scale bar, nm. *E*, X-ray diffraction of CRES3 seeds showed 4.6 and 10.6 Å reflections characteristic of amyloid.

Full-length mouse CRES3 expressed with a polyhistidine tag was isolated from bacterial inclusion bodies by nickel chromatography under denaturing conditions. Following dilution into aqueous buffer and adjustment to neutral pH, CRES3 immediately formed a white precipitate. The sample was centrifuged to further separate the soluble and insoluble fractions and both were examined by SDS-PAGE. As shown in the Coomassie-stained SDS-PAGE gel in Figure 1*A*, the majority of CRES3 was present in the insoluble (pellet, P) fraction and at the expected molecular weight (∼14 kDa) of a monomer suggesting that exposure to the 2% SDS in the gel loading buffer reversed higher-ordered structures. However, western blot analysis of a comparable sample separated on a gradient gel and incubated with an anti-CRES3 antibody showed that SDS stable forms with molecular weights suggestive of a dimer (∼28 kDa), tetramer (∼56 kDa), and larger forms were also present (Fig. 1*A*, right, arrows). A small proportion of CRES3 monomer was also detected in the soluble (supernatant, S) fraction (Fig. 1*A*). Dynamic light scattering revealed that the majority of the particles were much larger than the expected size for CRES3 monomer suggesting that even in the soluble fraction much of CRES3 had assembled into early aggregates (Fig. 1*B*). In support of this, in addition to granular material, individual ball-like structures typical of amyloid oligomers as well as protofibrils and occasionally early matrix were observed in the soluble fraction by negative stain TEM (Fig. 1*D*, supernatant). Circular dichroism (CD) of the soluble fraction indicated a protein with mixed secondary structure that was predicted, based on the BestSel program that is optimized for β-sheet-rich proteins (24), to contain primarily antiparallel β-sheets rather than the parallel β-sheets more commonly found in previously described amyloids (Fig. 1*C*). This observation, however, was not unexpected based on prior CD, FTIR, X-ray crystallization, and NMR studies of the related CRES that also revealed an antiparallel β-sheet-rich structure (25, 26).

**Figure 2:**
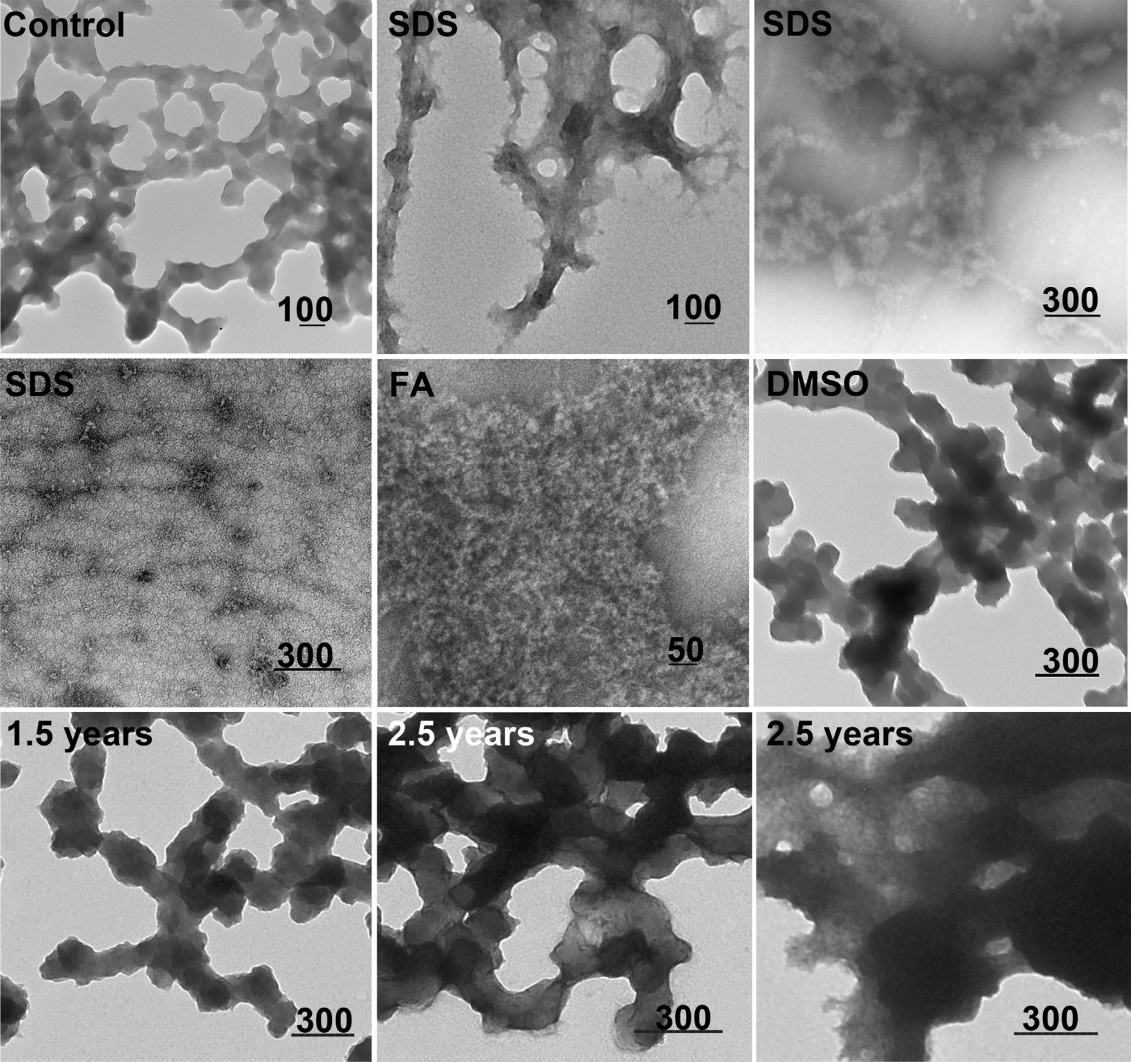
**Stability and structural changes in the CRES3 aggregates after exposure to different denaturants**. CRES3 pellet fractions containing beaded chains were exposed to 0.5% SDS for 20 min, 70% formic acid (FA) for 30 min, and 90% DMSO for 45 min, and structures examined by negative stain TEM. Control, CRES3 pellet incubated in 50 mM HEPES, 100 mM NaCl, pH 7.4 for 30 min. 1.5, 2.5 years, CRES3 beaded chains after storage in 50 mM HEPES, 100 mM NaCl, pH 7.4 at 4 °C for 1.5 and 2.5 years. Scale bar, nm.

In contrast to the heterogeneous soluble fraction, the insoluble fraction of CRES3 contained only networks of beaded chains (Fig. 1*D*, pellet). We previously showed that these CRES3 structures were highly reactive to ThT and the antifibrillar amyloid OC antibody suggesting that they possess cross-β-sheet architecture typical of amyloid (11). This was confirmed here by X-ray diffraction that yielded the characteristic cross-β reflections at 4.6 and 10.6 Å (Fig. 1*E*). The stability and structure of the CRES3 amyloid following exposure to different denaturants were examined by TEM. As shown in Figure 2, after incubation in 0.5% SDS, the CRES3 beaded chains partially disassembled into matrix, worm-like protofibrils, and oligomers indicative of SDS-resistant forms. Similarly, exposure to 70% formic acid resulted in the disassembly of the beaded chains to protofibrils while 90% DMSO had little effect. Taken together with our previous studies, these experiments show that CRES3 aggregates exhibit the properties of amyloid but assemble into large branched beaded chains that are unique from the typical amyloid fibril routinely studied. Further, once formed these structures are highly stable and do not transition into other morphologies even after prolonged storage (1+ year) at 4 °C. Only after 2+ years were some CRES3 beaded chains observed undergoing transition (Fig. 2). Similar branched beaded chains were detected by TEM in mouse epididymal luminal fluid following binding and elution from the protein aggregation disease (PAD) ligand, which binds cross-β-sheet structures, suggesting that these amyloid structures also function *in vivo* (Fig. 1*D*, epididymis).

### Solid-state NMR suggests that β-sheet transitions in CRES3 amyloid are similar to that in CRES

**Figure 3:**
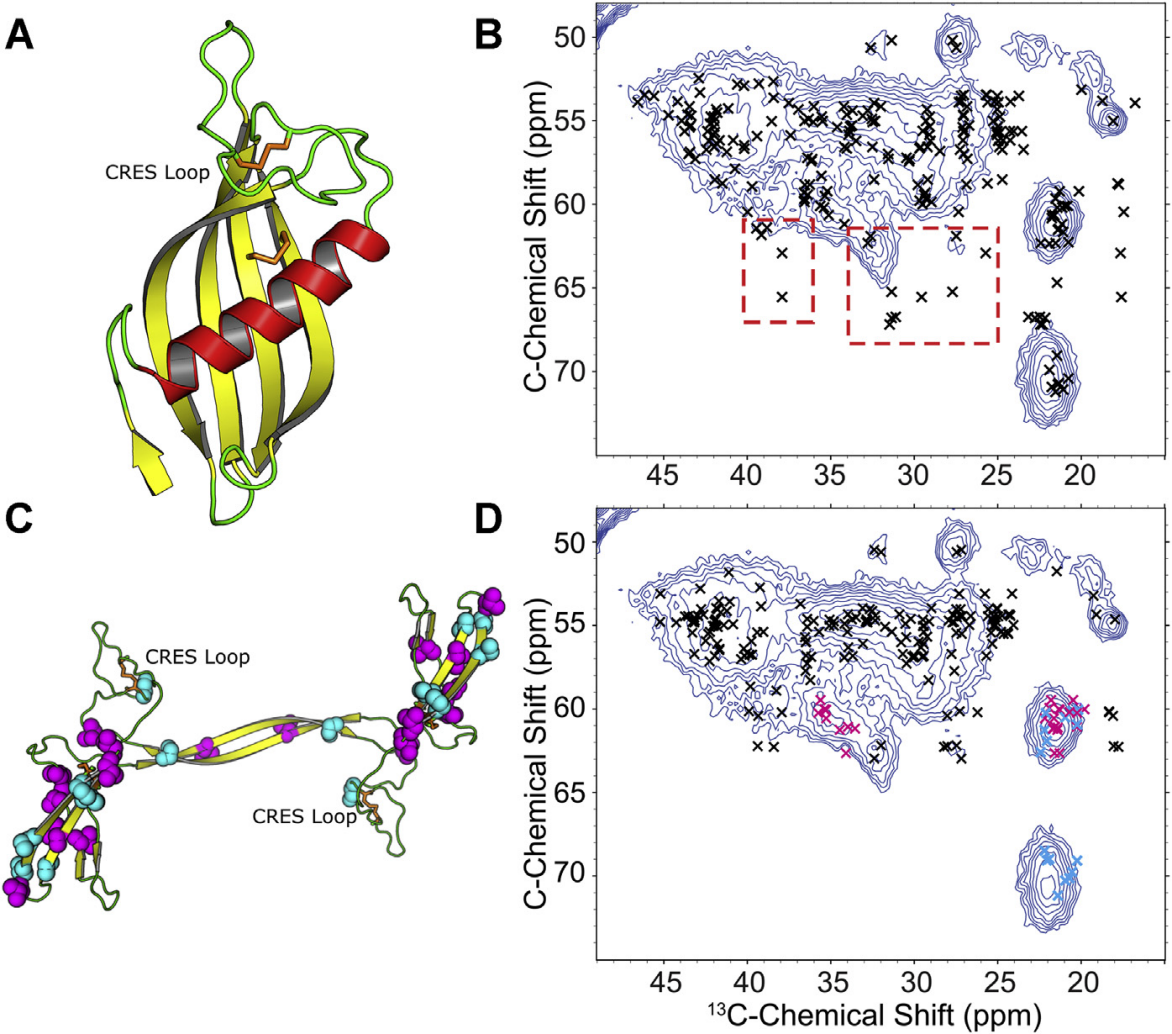
**Two-dimensional solid-state NMR ^13^C-^13^C spectrum of CRES3 analyzed with the aid of chemical shifts predicted from two homology models.***A*, structural model for the CRES3 monomer based on the X-ray crystal structure of CRES (pdbcode:6UIO) (26). A sequence alignment of mouse CRES, CRES2, CRES3, cystatin E2, and cystatin C was used to compute a homology model with PROMALS3D (28) and Modeller (29). Disulfide bonds are indicated in orange. *B*, chemical shifts (indicated by x) for the homology model were predicted with SHIFTX-2, converted into ^13^Cα-^13^C side-chain peak lists using FANDAS, and overlaid onto a 2D DARR (12 ms) ^13^C–^13^C correlation spectrum of U-^15^N,^13^C-CRES3 (*blue*). The predicted chemical shifts for β-sheet and turn residues in the protein agree well with the DARR spectrum. However, chemical shifts predicted from the α-helix (*red* in [*A*] and *red boxes* in [*B*]) agree poorly with the experimental spectrum. *C*, structural model for CRES3 with only β-sheet and turn geometries allowed. ^13^C labeled sites for Thr and Val shown in cyan and magenta, respectively. *D*, predicted cross-peaks from the structure in (*C*) agree well with the experimental spectrum, indicating β-sheet, and not α-helical structure is largely present. This includes the canonical β-sheet chemical shift correlations for Thr ^13^Cα-^13^Cγ2 and Thr ^13^Cβ-^13^C γ2 (*cyan* coloring of predicted peaks) and Val ^13^Cα-^13^Cγ1,2 canonical β-sheet chemical shift correlations (*magenta* coloring of predicted peaks).

A 2D ^13^C–^13^C solid-state nuclear magnetic resonance (SSNMR) spectrum of the CRES3 beaded chains revealed ^13^Cα-^13^Cβ and ^13^Cα-^13^C_side chain_ correlations indicative of a highly β-sheet and turn rich protein as expected for an amyloid (Fig. 3, full DARR spectrum in Fig. S1, spectra deconvolution *via* TIDE depicted in Fig. S2). ^13^Cα, ^13^Cβ, and some ^13^Cγ (*via* favored rotameric conformational preferences among different secondary structures) chemical shifts are well-known reporters of secondary structure (27). In the absence of complete chemical shift assignments, we predicted ^13^C chemical shifts using two structural models and used these hypothetical ^13^C–^13^C cross-peaks to determine the dominant secondary structure in the recorded CRES3 spectra. In the first model, we assumed that monomeric CRES3 retains the globular fold of a cystatin. Using PROMALS3D (28) and Modeller (29), we computed a CRES3 model based on our recently reported X-ray crystallographic structure of CRES (Fig. 3*A*) (26). In the second model, we computed a 3D CRES3 structure using our proposed domain-swapped amyloid-core model based upon the complete U-^15^N,^13^C chemical shift assignments of CRES amyloid (Fig. 3*C*) (26). In the overlay of the ^13^C–^13^C cross-peaks predicted from the CRES structure (Fig. 3*B*), the outlying peaks in red boxes correspond to the conserved cystatin α-helix. The lack of helical secondary structure in the CRES3 spectrum suggests that the assembly of the CRES3 amyloid involves a transition of α-helix to β-strand, similar to conformational changes observed in the polymerization of other amyloids including CRES (26). We confirmed that this is a likely possibility by repeating this exercise using our β-sheet and turn only CRES3 model (Fig. 3*D*). Here, we observed much greater overall agreement between the predicted chemical shifts and the experimental data. This included the predicted chemical shifts from Thr and Val residues (colored in cyan and magenta, respectively) that are distributed throughout the four β-strands of the hypothetical CRES3 domain-swapped dimer, which overwhelmingly agree with our acquired CRES3 spectrum (Fig. 3, *C* and *D*). While it is highly unlikely that this dimeric structure appears in the oligomeric CRES3 amyloid structure, it is clear that the β-sheet-rich secondary structural elements depicted in Figure 3*C* predominate.

Our experimental spectra indicate that CRES3 possesses structurally and dynamically heterogeneous quaternary structure. Thus, we leveraged a recently developed processing technique, T2∗ weIghted DEconvolution (TIDE), to deconvolute and better analyze our ^13^C–^13^C spectra (30). TIDE processing improves upon conventional 2D ^13^C–^13^C covariance processing by introducing a pseudo third dimension, which encodes the effective transverse relaxation (T2∗) dispersion among observed ^13^C resonances. The increased signal-to-noise ratio afforded by TIDE processing confirms that no residual Cα-Cβ α-helical chemical shifts are observed in the spectra of CRES3 (Fig. S2). Further, several random coil chemical shift correlations for Ile Cα-Cγ1 are observed and enhanced in the TIDE processed data. One of these outlier peaks may be uniquely assigned to the I14 Cα-Cγ1 correlation located near the hypothetical α-helix that would exist in the purported CRES3 globular fold. The T2∗ of this peak appears to be longer-lived than many other isoleucine resonances. A peak proposed to be this resonance appears strongly in TIDE planes corresponding to resonances with longer T2∗ (Fig. S2). This secondary structure and the relative dynamics suggested by the longer T2∗ are consistent with the changes in dynamic structure we reported for the CRES protein after it transitioned to its amyloid form (26). Our previous X-ray crystallographic and SSNMR studies of CRES also revealed that a flexible disulfide-anchored loop, which we call the CRES loop, transitioned to β-strand during amyloidogenesis (26). Because CRES3, like CRES, possesses the four cysteine residues that form the highly conserved disulfide bonds in family 2 cystatins, it also has a disulfide-anchored CRES loop (Fig. 3*A*) (11, 21). As shown here, several peaks in or near the purported CRES loop exhibit well-defined, rigid secondary structure in CRES3 (Fig. S2, *A* and *B*). These resonances include two prolines (72 and 77) in CRES3, which are clear chemical shift outliers exhibiting shorter T2∗, indicative of greater rigidity and packing in SSNMR spectra (because there is less dynamic averaging of ^1^H-^13^C dipole–dipole couplings). Both proline Cβ chemical shifts indicate β-sheet secondary structure, supporting the hypothesis that the CRES loop in CRES3 also adopts a largely β-sheet secondary structure within the CRES3 amyloid. As expected, the TIDE processing of these two proline Cδ-Cβ and Cδ-Cγ correlations exhibits a dramatic reduction in signal intensity between the planes corresponding to 0.2 and 0.6 of the T2∗ envelope, respectively (Fig. S2 compare A with B). These are clear examples illustrating how the dynamic timescales of different regions of the protein are dramatically different within the complex amyloid structure. Further, the profound deconvolution witnessed in the TIDE processing confirms that the predicted secondary structure in the amyloidogenic form of CRES3 agrees strongly with the observed spectra.

### CRES3 self-seeding

**Figure 4:**
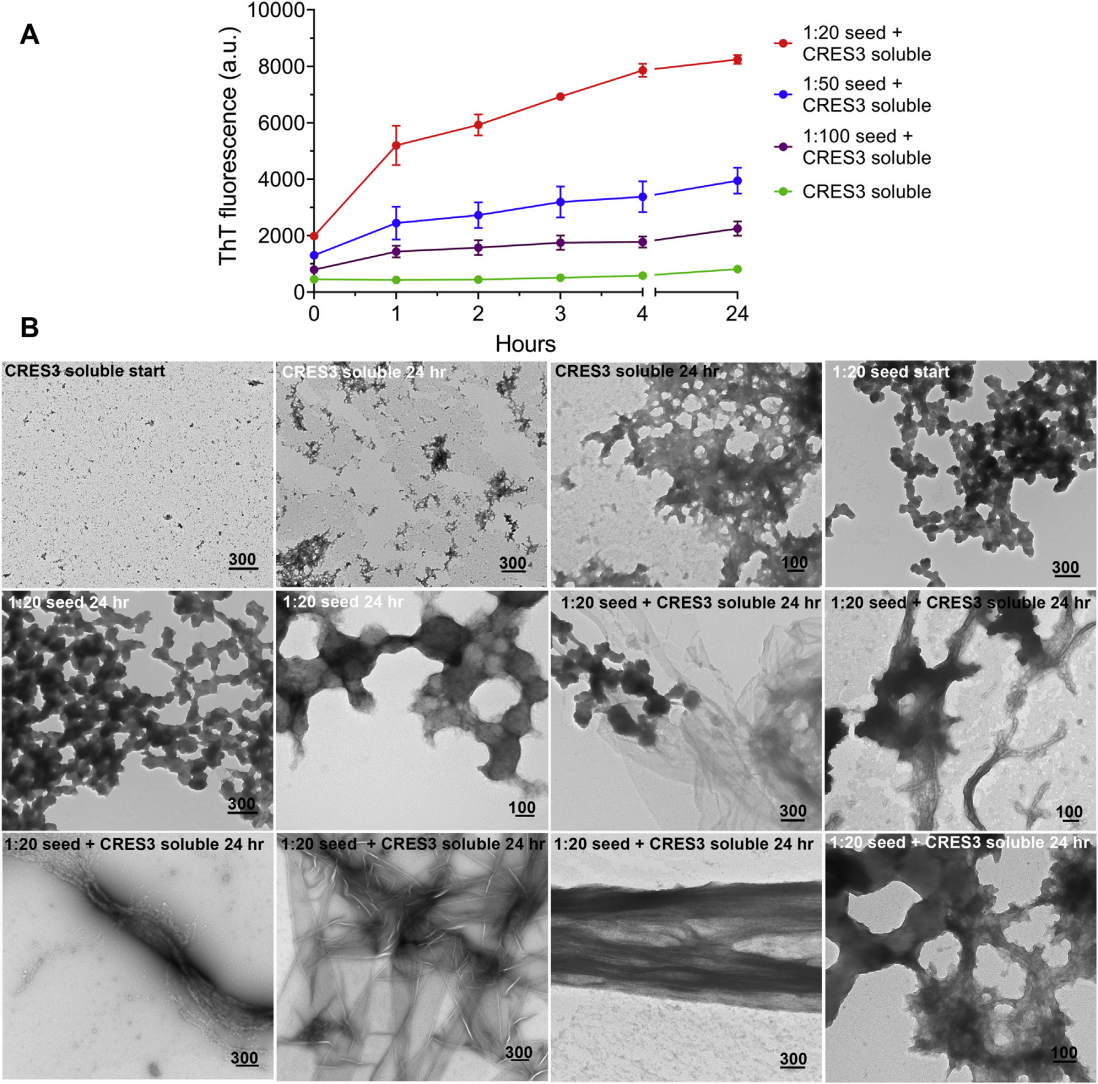
**Self-seeding of CRES3**. *A*, different dilutions of CRES3 seeds (1:20, 1:50, 1:100) were added to 10 μM CRES3 soluble fraction and assembly of amyloid monitored over time using a ThT plate assay. After 4 h the plates were sealed to prevent evaporation and then read again after 24 h total incubation. Data are presented as mean ± SEM of three independent experiments using three different CRES3 protein preparations. The ThT values for the individual seeding reactions are shown in Figure S3. The error bars for the CRES3 soluble fraction are too small to be detected. *B*, negative stain TEM of the CRES3 1:20 ThT seeding reactions after 24 h. Start, samples of the CRES3 soluble fraction and seed before incubation with ThT. Scale bar, nm.

We next used ThT plate assays to determine if the CRES3 beaded chain amyloids could function as a seed to facilitate amyloid assembly in the CRES3 soluble fraction. Preclearing the soluble fraction to remove existing oligomers was unsuccessful since very little protein remained and therefore whole soluble fractions were used. As shown in Figures 4*A* and S3, the addition of preformed CRES3 beaded chain seeds to the CRES3 soluble fraction resulted in an immediate and dose-dependent increase in ThT fluorescence indicative of increased amyloid assembly in the seeded sample compared with the unseeded soluble fraction or seed alone. The absence of a lag phase, including with the lowest dose of CRES3 seeds (1:100), is distinct from many previously described seeding reactions and suggests the presence of abundant preformed nuclei that are immediately capable of functioning as templates (31). The CRES3 soluble fraction alone sample exhibited little change in ThT fluorescence over the time course (Fig. 4*A*). While we cannot rule out the possibility that some soluble CRES3 is trapped in a nonamyloidogenic conformation, its slow assembly likely reflects the low concentration used in the ThT assay. A low concentration (10 μM) was used to optimize detection of ThT fluorescence resulting from the seeding reaction. Amyloidogenesis was followed over 4 h, and then the plate was sealed and stored overnight to prevent evaporation and then read the following day after a total of 24 h incubation. The ThT reactions at 24 h were also examined by TEM to visualize changes in amyloid conformation. The CRES3 soluble fraction alone after 24 h showed a slight increase in the amount of higher-ordered structures as evidenced by the occasional presence of early matrix assemblies (Fig. 4*B*). The CRES3 seed alone showed little or no change after 24 h. Only occasionally a beaded chain appeared that may be partially disassembling, possibly as a result of being diluted in the final reaction volume. In contrast, soluble CRES3 seeded with CRES3 beaded chains exhibited a remarkable change in structure dominated by the presence of amyloid films and some fibrils (Fig. 4*B*). The majority of the beaded chains had disappeared, and the few that remained were in some state of transition merging into films, bundles of films/fibrils, or sometimes were associated with matrix. Some films had dark centers that we speculate may be a remnant of a bead. These studies suggest that the CRES3 beaded chains, or components of the beaded chain, function as a nucleator to facilitate amyloid assembly in monomer and early assemblies of CRES3.

### Cross-seeding between CRES3 and CRES

**Figure 5:**
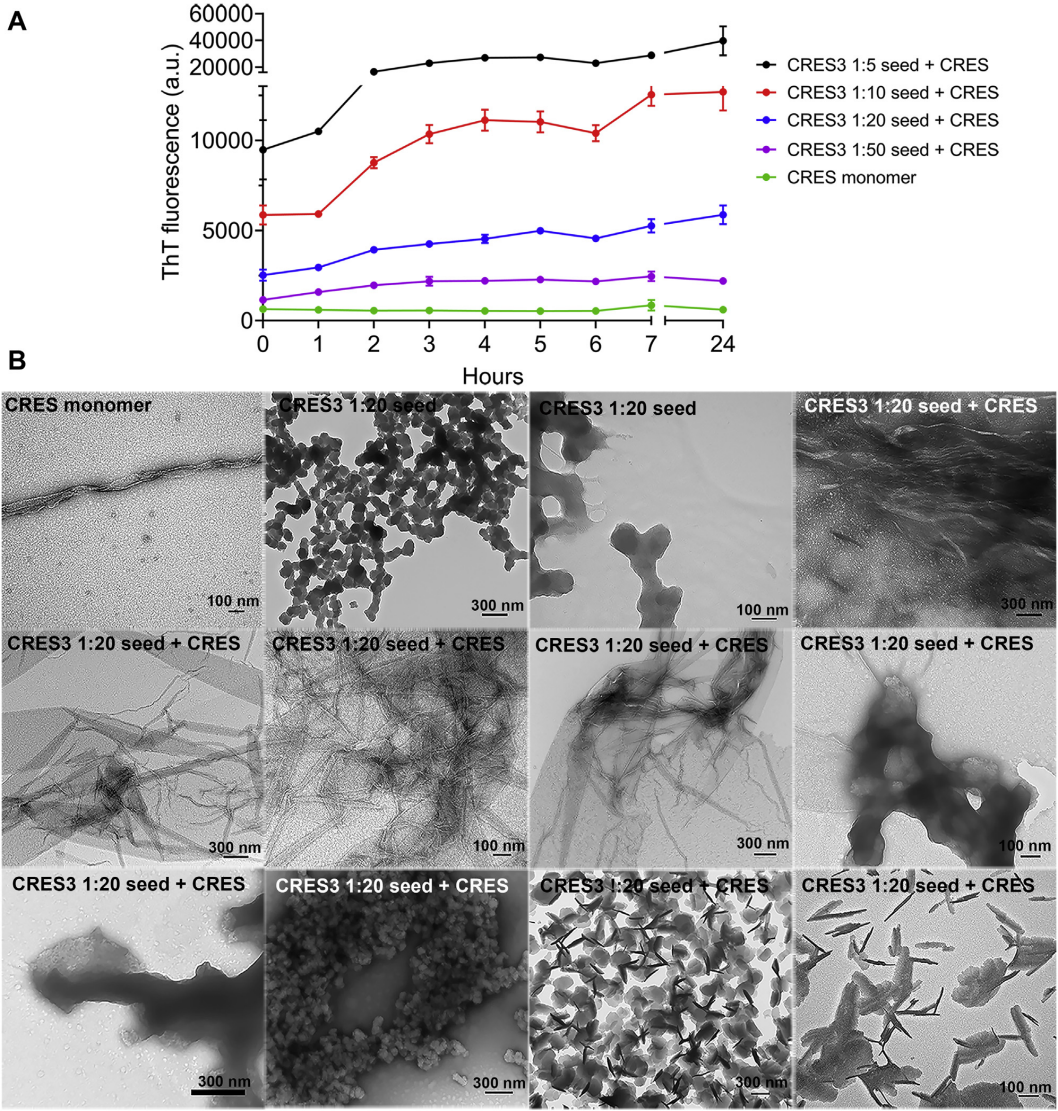
**CRES3 beaded chains template amyloid assembly in CRES C48A monomer.***A*, different amounts of CRES3 seeds (1:5, 1:10, 1:20, 1:50) were added to 10 μM CRES C48A monomer and assembly of amyloid monitored over time using a ThT plate assay. After 7 h the plates were sealed to prevent evaporation and then read again after 24 h total incubation. The data are presented as the mean ± SEM of three independent experiments using three different CRES3 and CRES C48A preparations. The error bars for the CRES C48A monomer are too small to be detected. The ThT values for the individual seeding reactions are shown in Figure S4. *B*, negative stain TEM of the ThT CRES3 1:20/CRES C48A seeding reactions after 24 h. Scale bar, nm.

We next determined if the CRES3 seeds could template assembly of the related amyloidogenic precursor CRES. Because CRES is less aggregation-prone than CRES3 and other subgroup members, in previous studies we were able to isolate a tagless full-length mouse CRES C48A under nondenaturing conditions from the soluble fraction of bacteria and follow its assembly to amyloid (25). The free cysteine (C) at position 48 was mutated to alanine (A) to prevent improper disulfide bond formation during purification. These studies showed that under physiological conditions, CRES C48A rapidly assembled from its natively folded monomer into an early oligomer that, if kept at high concentrations, remained stable (25). Only with extended time oligomers slowly transitioned into higher-ordered amyloids (25). From these studies we hypothesized that *in vivo* the CRES oligomer may function as a stable building block for the assembly of the highly branched and elaborate amyloid matrix that is present in the mouse caput epididymal lumen. For this reason, CRES might require a trigger to initiate its assembly into higher-ordered amyloids. As shown here, the addition of CRES3 seeds to CRES C48A monomer caused an immediate and dose-dependent increase in ThT fluorescence above that observed in CRES C48A monomer or CRES3 seed alone (Figs. 5*A* and S4). Similar to CRES3 self-seeding, no lag phase was observed. TEM of the samples after 24 h revealed an almost complete loss of the CRES3 beaded chains in the CRES C48A seeded sample that correlated with the appearance of an abundance of films, similar to that observed in the CRES3 self-seeding experiments. However, other structures were also detected in the cross-seeded samples including stacks of short fibrils (Fig. 5*B*). CRES C48A monomer alone after 24 h showed primarily granular and small particulate structures and sometimes short fragments of film or early matrix as previously described (Fig. 5*B*) (25).

**Figure 6:**
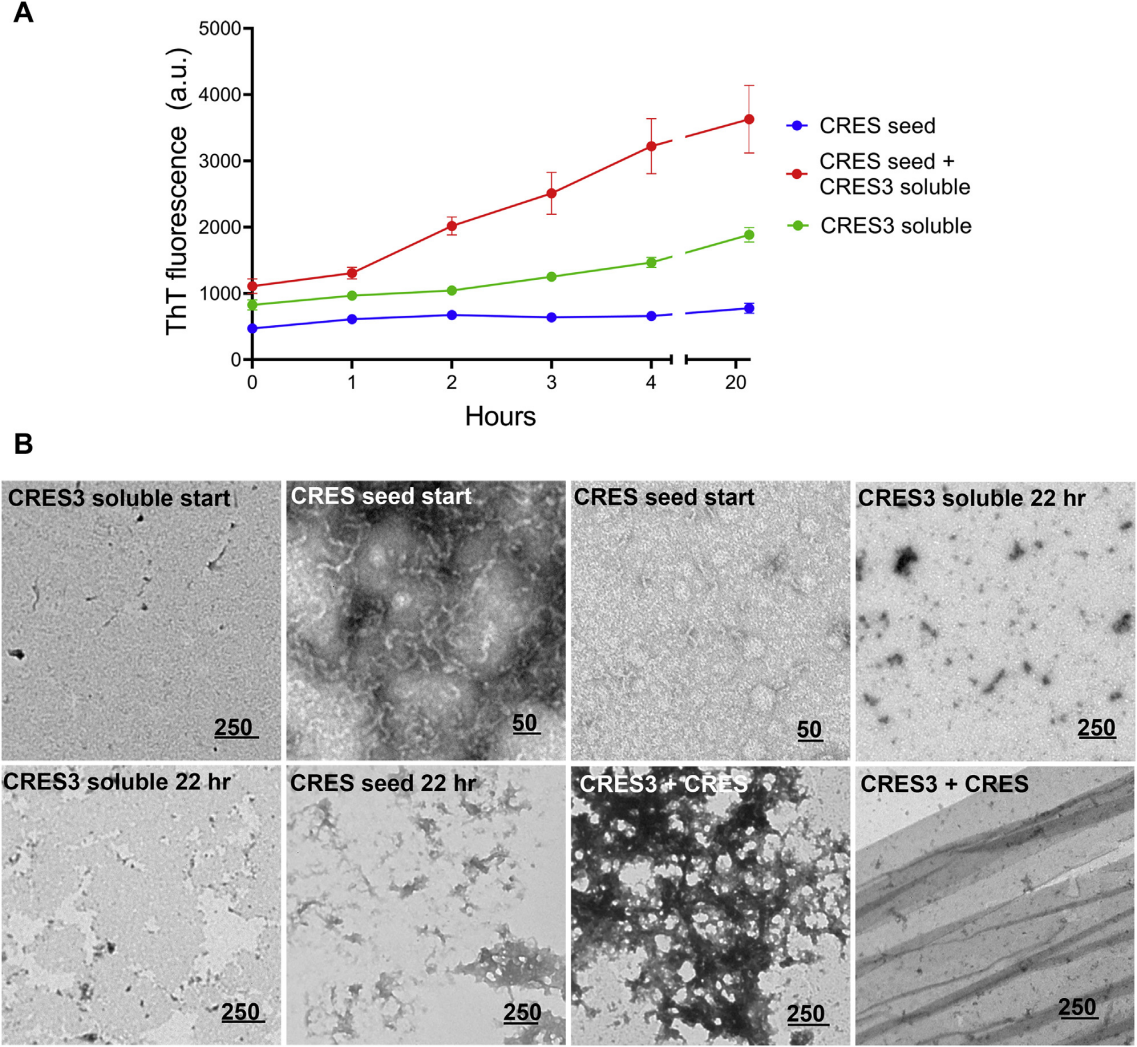
**CRES seeds template amyloid assembly in the CRES3 soluble fraction.***A*, CRES seeds (1:20) were added to 10 μM soluble CRES3 and assembly of amyloid followed with time by ThT fluorescence. After 4 h the plates were sealed to prevent evaporation and read again after 22 h total incubation. Data shown are the mean ± SEM of three independent experiments using 2–3 different CRES and CRES3 preparations. Error bars for CRES seed and CRES3 soluble fraction alone are too small to be detected. *B*, negative stain TEM of the CRES3 soluble and CRES seed samples alone at time 0 (start) and after 22 h and of the CRES-CRES3 seeded samples after 22 h incubation at RT. Scale bar, nm.

We also carried out the opposite experiment in which CRES seeds were added to the CRES3 soluble fraction. Because CRES C48A early oligomers generated from natively folded protein lacked sufficient cross-β structure to be an effective seed, we used CRES expressed with a polyhistidine tag and isolated from bacterial inclusion bodies as seed in these experiments. Unlike the beaded chains in the CRES3 seed, CRES seed alone at time 0 showed a predominance of oligomers and protofibrils that after 22 h had started to assemble into small patches of matrix, typical for CRES when it does transition into higher-ordered amyloid (25). Although the CRES seeds facilitated amyloid assembly in the CRES3 soluble fraction, the overall increase in ThT fluorescence was not as profound as that observed when CRES3 seed was added to CRES monomer; further there was a noticeable lag of ∼1 h (Fig. 6*A*). The presence of a lag phase in the CRES-CRES3 cross-seeding suggests that conformational changes in the CRES seed were necessary, which were not required with CRES3 seed (beaded chains), to template assembly. TEM showed that after 22 h the addition of CRES seeds to CRES3 resulted in an abundance of amyloid matrix with some films (Fig. 6*B*). The predominance of amyloid matrix suggests the CRES seed effectively templated CRES3 into CRES’ primary conformer rather than forming films and fibrils as observed when the CRES3 beaded chains were the seed. Taken together our experiments show that cross-seeding can occur between the CRES subgroup of mammalian amyloidogenic proteins.

### CRES and CRES3 regulate Aβ amyloid assembly

**Figure 7:**
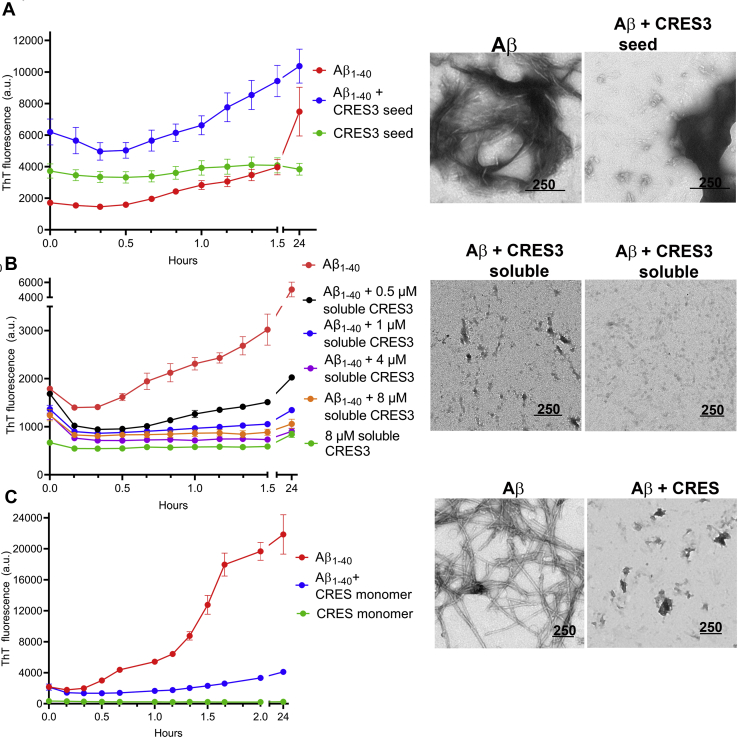
**CRES3 and CRES regulate Aβ**_**1-40**_**amyloidogenesis.***A*, CRES3 seed (1:20) or (*B*) 0.5–8 μM soluble CRES3 was added to 8 μM Aβ and ThT fluorescence determined over 1.5 h. The plates were sealed and then read again after 24 h. Data shown are the mean ± SEM of 9–12 replicates from three independent experiments. The ThT values for the individual soluble CRES3/Aβ reactions are shown in Figure S5. Aliquots of the ThT reactions were examined by TEM after 24 h. Additional TEM images are shown in Figures S6 and S7. *C*, 8 μM CRES C48A monomer was added to 8 μM Aβ and ThT fluorescence determined over 2 h. The plates were sealed and then read again after 22 h. Data shown are the mean ± SEM of six replicates from two independent experiments. Additional TEM images are shown in Figure S8. (*A–C*), Scale bar, nm.

Studies *in vitro* and *in vivo* have shown that unrelated amyloidogenic proteins can cross-seed promoting amyloid assembly in one another (32, 33, 34). These include interactions between human tau, Aβ, α-synuclein, and PrP, as well as across kingdoms between bacterial curli and human α-synuclein (32, 33, 34, 35). Aβ, in particular, interacts with several different amyloidogenic precursors (36). Further, amyloid precursor protein (APP), the precursor to Aβ, and the related APLP2 are expressed in the testis and epididymis and important for fertility suggesting that APP/Aβ/CRES subgroup cross-seeding interactions could occur (37, 38, 39, 40). Therefore, CRES3 seed was added to Aβ_1-40_ and amyloidogenesis followed by ThT fluorescence and TEM. Although the addition of CRES3 beaded chains to Aβ did not cause an increase in ThT fluorescence above that present in the combined fluorescence from Aβ and CRES3 seed alone (Fig. 7*A*), TEM revealed that changes in protein structure had indeed occurred and appeared to be time-dependent (Figs. 7*A*, S6, and S7). We believe our inability to detect a change in ThT in the seeded sample may be due to the already high levels of ThT fluorescence in the individual CRES3 seed and Aβ samples. TEM showed that after 1.5 h the typical long fibrils and fibrillar tangles present in Aβ alone were absent in the CRES3-Aβ seeded reactions (Figs. 7*A* and S6). However, patches of short fibrils were observed in the seeded samples (which also were detected in Aβ alone) as were small fragments of film suggesting that the CRES3 seeds may have only slowed down Aβ assembly (Fig. S6). Indeed, after 24 h most CRES3 beaded chains now had matrix and fibrils that were assembling from discrete points of individual beads (Fig. S7). We speculate that this represents Aβ using the CRES3 bead as a surface/attachment point for polymerization. In contrast to CRES3 seed, the addition of soluble CRES3 prevented, in a dose-dependent manner, the assembly of Aβ amyloid even after 24 h incubation (Figs. 7*B* and S5). Not only was ThT reduced to levels similar to that in the CRES3 soluble fraction alone but TEM showed that the Aβ amyloid fibrils and films were now noticeably absent after 1.5 and 24 h (Figs. 7*B*, S6, and S7). We observed similar results when CRES monomer was incubated with Aβ (Figs. 7*C* and S8). Together these results show that CRES3 seed can template the unrelated amyloidogenic precursor Aβ and suggest that CRES subgroup members may regulate Aβ amyloidogenesis by both amyloid-promoting and amyloid-inhibiting activities.

Lastly, these experiments showed that the polymerization of Aβ shared features with that of CRES3. In addition to fibrils, after 1.5 h some Aβ had assembled into large beads that contained worm-like structures, which we usually observed merging into films, although individual but intact beads without film were also sometimes detected (Fig. S6). Although the Aβ beads did not form chains as in CRES3, they may be functionally similar and be units of preassembled oligomers. Whether the beads and films represent Aβ in an earlier transition state along the same assembly pathway, or alternatively, a different assembly pathway than to fibril is not known.

## Discussion

### Cross-seeding as a conserved mechanism to control the assembly of biological amyloids

Because nature has elected to keep the amyloid fold, its benefits must outweigh its risks to the organism. This is evidenced by the rapidly growing number of biological amyloids that have been described including in human (3, 8, 41). Although mechanisms to control functional amyloid assembly are essential to prevent pathology, they remain poorly understood. With our studies here of full-length mouse CRES3 and CRES, we show that cross-seeding between amyloidogenic family members occurs in mammals similar to that which has been described in bacteria (16). Like the curli proteins in *E. coli*, cross-seeding between CRES subgroup members may be a mechanism to control the formation of a functional extracellular amyloid. Therefore, in addition to keeping the amyloid conformation for biological purposes, nature may have also preserved a mechanism for regulating its assembly.

In particular, our studies show that CRES3 aggregates function as nucleators to facilitate amyloidogenesis in CRES and may perform similar functions for other subgroup members. The presence of a preassembled seed would allow an expedited assembly to higher-ordered amyloids, not only limiting exposure to potentially toxic oligomers but also allowing the formation of heterotypic amyloids that may be integral for the biological function(s) of the epididymal amyloid matrix. The beaded chain appearance of the CRES3 seed is also distinct from most previously described amyloid conformations; whether this is significant for its role as a nucleator requires further study. However, as shown here, the presence of similar beaded chain amyloids in the mouse epididymal lumen and in our previous studies in isolated mouse zona pellucidae (ZP) after exposure to chymotrypsin, which exposes the ZP amyloid fibrils, suggests that these unique structures perform biological roles (4). Our experiments also showed that the CRES3 beaded chains are highly stable and required harsh denaturants for their partial disassembly. However, when added to a soluble fraction of CRES3 or CRES, the beads quickly disappeared and films, with some fibrils, became the dominant structure. Together, these results suggest that the CRES3 beads may function as stable preassembled early amyloids (seeds) that can rapidly template the polymerization of higher-ordered amyloids on an as-needed basis.

### Complexity of functional amyloid assembly

Our studies of the CRES3 beaded chain amyloids revealed different seeding behaviors depending on the amyloidogenic precursor with which they interacted. While the beaded chains disassembled and formed new structures when templating soluble CRES3 or CRES monomer, with Aβ the CRES3 beaded chains remained intact and served as attachment points for Aβ fibril formation. In this regard, our results are similar to that observed in cross-seeding experiments between the prion domain of yeast prion Rnq1 and the yeast prion sup35NM (23). In these studies, the Rnq1 bead, referred to as a spherical seed by the authors, also served as a surface on which the sup35NM fibrils assembled resulting in a hybrid structure of sphere and fibril (23). While we cannot eliminate the possibility that with extended time the CRES3 seeds might also become a part of an Aβ fibril, our results suggest that there are two different mechanisms by which the beaded chains can enable amyloid assembly. The two distinct ways of seeding by CRES3 may reflect whether the interactions are between related or unrelated proteins, the relative ratios of the two proteins, their structures, or some other variable. Functionally these different seeding mechanisms could be equivalent to β-sheet interactions that form the infrastructure of a scaffold and β-sheet interactions that allow independent assemblies to attach or anchor to the scaffold, both of which may be necessary for the controlled assembly of biological amyloids with intricate quaternary structures.

Our studies with Aβ show that CRES subgroup members exhibit both amyloid-promoting and amyloid-inhibiting functions. As we observed with CRES3, both properties can occur within the same protein depending on its structure. While the CRES3 beaded chains promoted Aβ assembly, soluble forms of CRES3, and CRES, inhibited Aβ amyloid formation. Similarly, previous reports showed that the related cystatin C inhibited Aβ amyloid assembly *in vitro* and further inhibited Aβ deposition in mouse models of Alzheimer’s disease suggesting that it plays a protective role in regulating Aβ amyloidogenesis and in disease progression (42, 43, 44). Subsequent studies showed specifically that a stable cystatin C oligomer rather than monomer inhibited Aβ assembly, possibly as a result of the proteins sharing structural features (45). Because the soluble CRES3 and CRES samples likely contained both monomer and early oligomer, we cannot determine which conformer inhibited Aβ in our studies.

We speculate that inhibitory interactions between amyloidogenic precursors also serve as a means to control the polymerization of biological amyloids. Several of the functional amyloids that have been described are sophisticated structures composed of several to many amyloidogenic precursors, often family members. In addition to the CRES subgroup/cystatin C in the epididymal amyloid matrix, three zona pellucida (ZP) domain containing proteins interact to form the ZP, a protective extracellular amyloid matrix that surrounds the oocyte and participates in fertilization (4, 46). Similarly, the sperm acrosomal matrix, which also plays a role in fertilization, contains multiple proteins that are known or predicted to be amyloidogenic (5, 47). The human RIP1 and RIP3 independently form amyloids but hetero-oligomerize to form a functional amyloid signaling complex that mediates programmed necrosis (41). It is likely that both amyloid-promoting and amyloid-inhibiting interactions between various family members, and possibly unrelated amyloidogenic precursors as well, may be needed for the formation of these biological amyloids ensuring they form at the right time and place and with the correct partners. In support this may be so, in *E. coli* the curli protein CsgC prevents CsgA amyloid assembly prior to its secretion and interaction with CsgB in the extracellular space (48). While our studies here showed that CRES and CRES3 facilitated amyloidogenesis with one another, experiments with the remaining subgroup family members CRES2 and cystatin E2 as well as the related cystatin C are needed to determine if any exhibit amyloid inhibitory activity toward other family members.

Finally, the atypical kinetics of CRES3 self-seeding and cross-seeding with CRES implies an additional level of complexity to CRES subgroup amyloidogenesis. Our NMR and X-ray crystallization studies of CRES revealed two mechanisms by which amyloid assembly occurs; a unique conformational switch of a highly flexible disulfide-anchored loop (CRES loop) to a rigid β-strand and by traditional cystatin domain swapping (26). Although further studies are required, our SSNMR data here suggest that both mechanisms may also contribute to CRES3 amyloidogenesis. It is possible that the unique properties of CRES3 seeding could reflect differential utilization of these assembly mechanisms. We have yet to establish the biological significance of multiple assembly mechanisms within one amyloidogenic precursor. However, they may provide a means to build highly branched amyloid matrices, including those with other amyloidogenic precursors, from a central amyloid core, and/or allow the disassembly of some β-sheet assemblies while still maintaining others. Both may be integral for the assembly and biological function of the epididymal amyloid matrix.

## Experimental procedures

### Animals

CD1 male mice were purchased from Charles River. Mice were maintained under a constant 12 h light/12 h dark cycle with food and water *ad libitum*. All animal studies were conducted in accordance with the NIH Guidelines for the Care and Use of Experimental Animals using a protocol approved by the Texas Tech University Health Sciences Center Institutional Animal Care and Use Committee.

### Isolation of epididymal luminal fluid and amyloid matrix

Caput luminal fluid and epididymal amyloid matrix were isolated from 26–31 week CD1 mice as described (11). Proteins were quantitated using the BCA assay (ThermoScientific). For PAD pull-down experiments, 200 μl of caput epididymal fluid (∼3–4 mg/ml) was added to the PAD beads following the manufacturer’s protocol (11). After binding at RT for 2 h on a rotator, PAD beads were captured with a magnet, washed, and bound proteins were eluted in 1× Laemmli buffer without reducing agents or bromophenol blue at RT for 5 min followed by 95 °C for 5 min. Samples were spotted onto grids for negative stain TEM as described below.

### Expression and purification of His-CRES3 and His-CRES

Recombinant 6× His-tagged full-length mouse CRES3 (*Cst12*) (Uniprot # Q9DAN8) and CRES (*Cst8*) (Uniprot # P32766) proteins were expressed in *E. coli* and isolated from inclusion bodies in 6 M guanidine-Cl, 25 mM MES (2-(4-morpholino)-ethane sulfonic acid) as previously described (49).

Following elution from the nickel NTA agarose column in 6M guanidine-Cl, 25 mM MES, pH 4.5, CRES3 was diluted 1:100 with 50 mM HEPES, 100 mM NaCl, pH 7.4, resulting in its aggregation. The sample was centrifuged at 171,500*g* for 1 h, 4 °C to generate the CRES3 soluble fraction (supernatant) and the seed (pellet), and both were stored at 4 °C. Immediately prior to analysis, the CRES3 soluble fraction was concentrated to 0.2–0.7 mg/ml using a 10 kDa cutoff Amicon filter (Millipore), exchanged into gel filtration buffer (25 mM MES, 250 mM NaCl, 1 mM EDTA, pH 6) or 4 mM potassium phosphate buffer pH 7.4 with PD10 columns (Sephadex G-25M, GE Healthcare), and used in ThT assays or for circular dichroism and dynamic light scattering, respectively. The CRES3 pellet was resuspended in 0.5 ml 50 mM HEPES, 100 mM NaCl, pH 7.4 and used as seed in ThT assays. CRES3 protein concentration in the pellet was assumed to be 100% of the starting protein concentration (determined after elution off the nickel column). The resuspended seed was diluted 1:5 in HEPES/NaCl buffer and gently sonicated 3 × 10 pulses at 20% duty using a Heat Systems Ultrasonic sonicator (Qsonica) to break apart large aggregates resulting in a homogeneous suspension. The CRES3 seed was used directly or further diluted immediately prior to use in ThT assays as described below.

His-CRES eluted off the nickel column was dialyzed using a 3.5 kDa cutoff, Spectra/Por 6 dialysis tubing (Spectrum Laboratories) to remove guanidine-Cl. The dialysis process was performed at 4 °C in buffers at pH 5.0 starting with 25 mM MES, 1 M guanidine-Cl for 24 h then 25 mM MES, 0.1 M guanidine-Cl for 24 h, and finally 25 mM MES for 24–48 h. His-CRES seeds were prepared by using a 30 kDa Amicon Ultra-15 filter (Millipore) to isolate the fraction that did not go through the filter. The protein sample was then concentrated to 6.5–7.3 mg/ml using a 10 kDa Amicon Ultra-15 filter (Millipore) and kept at 4 °C for 14–16 days before use for seeding experiments. Protein concentrations for CRES and CRES3 were determined using a Nanodrop Lite spectrophotometer (ThermoScientific) with the extinction coefficients 16,740 M^−1^ cm^−1^ for His-CRES (MW 15.45 kDa) and 12,330 M^−1^ cm^−1^ for His-CRES3 (MW 14.12 kDa).

### Preparation of CRES monomer

Tag-less CRES C48A modified with a cysteine 48 to alanine change (C48A) to prevent inappropriate disulfide bond formation during protein expression was purified from the soluble fraction of bacteria using affinity, ion exchange, and gel filtration chromatography as previously described (25). Protein concentration was determined using a Nanodrop with an extinction coefficient of 16,960 M^−1^ cm^−1^ calculated based on CRES residues 20V–142V and a six-residue N-terminal linker, GAMAHM. Immediately prior to the ThT experiments, CRES, stored at 4 °C at ∼ 0.15 mg/ml or less in gel filtration buffer, was centrifuged through an Amicon Ultra-15 30 KDa filter at 2500*g* 4 °C to remove existing aggregates. The filtrate was collected and concentrated to 0.33 mg/ml using an Amicon Ultra-15, 10 KDa and centrifugation at 2500*g*, 4 °C. This sample was kept on ice and used in seeding assays within 2 h after it was generated. We have previously demonstrated that this preparation contains monomeric CRES with some oligomeric species (25).

### Seeding experiments

The effect of preformed seeds on CRES and CRES3 aggregation was followed over time using ThT plate assays as previously described (25). Briefly, amyloid formation was monitored in 96-well black flat bottom plates (Corning) using a TECAN Infinite M1000 PRO microplate reader in the fluorescence top reading mode (Tecan). Amyloidogenesis was followed over 1.5 to 7 h, and then plates were sealed and stored overnight to prevent evaporation and then read the following day after a total of 24 h incubation. The excitation and emission wavelengths were 440 ± 5 nm and 485 ± 10 nm, respectively. All samples were done in duplicate or triplicate, and ThT fluorescence of the blank (reaction components without protein) was subtracted from each sample. For the CRES3 self-seeding experiments, different doses of seed were prepared by further diluting the sonicated CRES3 1:5 suspension (described above) (1:4, 1:10, and 1:20) in 50 mM HEPES, 100 mM NaCl, pH 7.4 immediately prior to the ThT assay, resulting in CRES3 seeds that were 1:20, 1:50, and 1:100 dilutions of the original CRES3 seed stock. Five microliters of each dilution were added to 10 μM CRES3 soluble fraction in 4 mM potassium phosphate pH 7.4 (final reaction buffer was 3 mM potassium phosphate, pH 7.4, 2.5 mM HEPES, 5 mM NaCl). ThT was added to 20 μM final and plates were read as described above. In the cross-seeding experiments, 5 μl of freshly diluted CRES3 seeds representing 1:5, 1:10, 1:20, and 1:50 of the original seed stock were added to 10 μM CRES monomer and 5 μl of CRES seeds (1:20) were added to 10 μM CRES3 soluble fraction in 50 mM HEPES, 100 mM NaCl followed by addition of ThT to 20 μM. ThT (Sigma, St Louis, MO) was prepared as a 0.1 mM stock in water, filtered, and stored in the dark at room temperature.

For seeding assays with Aβ, 8 μM Aβ was combined with 0.5–8 μM CRES3 soluble fraction in 0.5× PBS, 12.5 mM MES, 125 mM NaCl, 0.5 mM EDTA or 1:20 CRES3 seed in 0.5× dPBS, 1.5 mM potassium phosphate buffer, pH 7.4. Similarly 8 μM Aβ was incubated with 8 μM CRES monomer in 0.5× dPBS, 12.5 mM MES, 125 mM NaCl, 0.5 mM EDTA, pH 7.4. ThT was added to 20 μM and samples were immediately read.

### Preparation of Aβ

Aβ_1–40_ ultrapure, HCl (rPeptide) (0.5 mg) was dissolved in 100% DMSO to 2.5 mM and sonicated in a sonication bath for 10 min on high, aliquoted, and stored –20 °C until use. An 8 μl aliquot was thawed and diluted to 100 μM with cold IMDM media (Gibco/Invitrogen) briefly vortexed and stored overnight at 4 °C for use the next day in ThT assays.

### TEM

Starting proteins, samples taken directly from the ThT seeding experiments, or proteins eluted from PAD beads were spotted on to formvar-coated nickel grids and stained with 2% uranyl acetate as previously described (20).

### Circular dichroism

CRES3 in 4 mM potassium phosphate buffer, pH 7.4 was diluted to 0.2 mg/ml and examined using a J-815 CD spectrophotometer (JASCO Co). Spectra were acquired at 22 °C in the 190–260 nm spectral range at an acquisition rate of 1 nm/sec and a data pitch of 0.1 nm. Six CD spectra of each sample were averaged to calculate the final CD data. CD spectra were also measured for each buffer and were subtracted from the respective protein containing sample spectra. The secondary structure content of the samples was predicted from the spectral data using the BeStSel web server at http://bestsel.elte.hu/index.php with “input units” of measured ellipticity (mdeg), 113 residues of CRES3, and pathlength of 0.1 cm. Mean ± SEM was calculated from the predicted secondary structures of n = 3 independent CRES3 preparations.

### Dynamic light scattering

Soluble CRES3 in 4 mM potassium phosphate buffer pH 7.4 was examined using a Zetasizer Nano ZS (ZEN3600, Malvern Instruments) equipped with a 633 nm red laser and 173° scattering angle as described (25).

### SDS-PAGE and Western blot analysis

CRES3 was separated on a 15% or 4–20% Criterion Tris-glycine SDS-PAGE (BioRad Laboratories) and stained with Coomassie blue or transferred to PVDF (Immobilon-P) membrane (Millipore) and incubated with a rabbit anti-mouse CRES3 antibody (generated in house) in western blot analysis as described (11).

### Powder X-ray diffraction

CRES3 seeds in 50 mM HEPES, 100 mM NaCl, pH 7.4 were centrifuged at 5000*g* for 10 min to generate a pellet. The pellet was resuspended in 1 ml 5 mM ammonium acetate followed by centrifugation at 5000*g* to remove residual HEPES/NaCl. The final pellet was resuspended in 20 μl of 5 mM ammonium acetate, pH 7.3, and pulled into a 0.7 mm quartz capillary tube. The sample was allowed to air dry in the presence of desiccant. At 5 mM ammonium acetate is a volatile buffer and does not form crystals that would interfere with the diffraction pattern. X-ray diffraction data were acquired using a Rigaku Screen Machine (Rigaku) X-ray generator (50 kV, 0.6 mA) utilizing CuKα radiation (1.5418 Å) and mercury CCD detector. The distance from sample to detector was 75 mm. The sample was exposed to X-rays for 30 s.

### Solid-state NMR

Uniformly ^13^C, ^15^N-CRES3 was expressed in *E. coli* grown in M9 medium containing ^13^C-D-glucose (4 g/l) and ^15^N-amonium chloride (2 g/l) (Cambridge Isotope Labs) and supplemented with ^13^C–^15^N labeled Cell Tone Base powder (1 g/l) (Cambridge Isotope Labs). CRES3 purification is described above. The pellet containing CRES3 beaded chains was placed into a 90% humidity chamber of saturated KCl for 4 days at RT to slowly remove bulk water and then centrifuged at 16,000*g* 4 °C to collect the sample. This ^13^C–^15^N -CRES3 amyloid sample was packed into a 3.2 mm pencil rotor (Agilent Technologies). SSNMR data was acquired on a 600 MHz Agilent DD2 three-channel spectrometer equipped with an HCN Balun probe (Agilent Technologies). The magic-angle spinning rate and the sample temperature were maintained at 13.333 ± 0.002 kHz and at -5 ± 2 °C, respectively. ^13^C chemical shifts were externally referenced to adamantane. The downfield signal of adamantane was set to 40.48 ppm on the DSS scale. The two-dimensional DARR spectrum (50) was acquired employing 1 ms of ramped cross-polarization (51) and radio frequency fields of 60 kHz on ^13^C and 73 kHz on ^1^H. During cross-polarization, an 18% tangent ramp was applied to ^13^C. Data were acquired using 9 ms of ^13^C chemical shift evolution, 12 ms of DARR mixing, and 20 ms of directly detected acquisition. 85 kHz of SPINAL-64 (52) proton decoupling was applied during both chemical shift evolution periods. We processed the spectrum with NMRPipe (53) using 50 Hz Gaussian line broadening in both dimensions prior to Fourier transformation. The data was analyzed in NMRFAM-SPARKY (54, 55). A homology model for the CRES3 monomer was based on the X-ray crystal structure of CRES. A sequence alignment using mouse CRES(pdbcode:6UIO), CRES2 (Uniprot # Q9D269), CRES3 (Uniprot # Q9DAN8), cystatin E2 (Uniprot # Q9D264), and cystatin C (UniProt # P21460) was used to compute the homology model with PROMALS3D (28) and Modeller (29). The CRES3 homology model of the domain-swapped dimer was generated by SWISS MODEL (56, 57). Chemical shifts were predicted by SHIFTX-2 (58) and converted into peak lists using FANDAS (59).

TIDE processing was performed within MATLAB using code generously provided by V.S. Manu (30). The TIDE output was converted into NMRPipe format and then to NMRFAM-SPARKY format *via* in-house scripts and modified MATLAB code. TIDE pseudo-3D spectra were produced such that the envelope of the states-acquired t1 FID of the ^13^C-^13^C 2D spectra is normalized to one, with each increment of TIDE processing planes constituting 5% of the envelope (n). Increments were arrayed together such that each plane number (p) was p∗n of the t1 dimension. This is done in such a way that the coherence lifetime must be longer at smaller values of p∗n.

## Data availability

All data are contained within the article.

## Conflict of interest

The authors declare that they have no conflicts of interest with the contents of this article.
