# Supplementary material for: Cross-seeding between the functional amyloidogenic CRES and CRES3 family members and their regulation of Aβ assembly
Source: J Biol Chem. 2021 Jan 9;296:100250. doi: 10.1074/jbc.RA120.015307 (PMC7948811; doi:10.1074/jbc.RA120.015307)
Supplement: Figures S1–S8 [file mmc1.pdf]

Cross-seeding Between the Functional Amyloidogenic CRES and CRES3 Family Members and their Regulation of Aβ Assembly

Hoa Quynh Do[1], Aveline Hewetson[1], Collin G. Borcik[2], Mary Catherine Hastert[3], Sandra Whelly[1], Benjamin J. Wylie[2], Roger Bryan Sutton[4], Gail A. Cornwall[1*]

[1]Department of Cell Biology and Biochemistry, Texas Tech University Health Sciences Center, Lubbock, TX, 79430, [2]Department of Chemistry and Biochemistry, Texas Tech University, Lubbock, TX 79409, [3]College of Arts and Sciences Microscopy, Texas Tech University, Lubbock, TX, 79409, and [4]Department of Physiology and Molecular Biophysics, Texas Tech University Health Sciences Center, Lubbock, TX 79430.

Supporting information provided:

Figures S1-S8

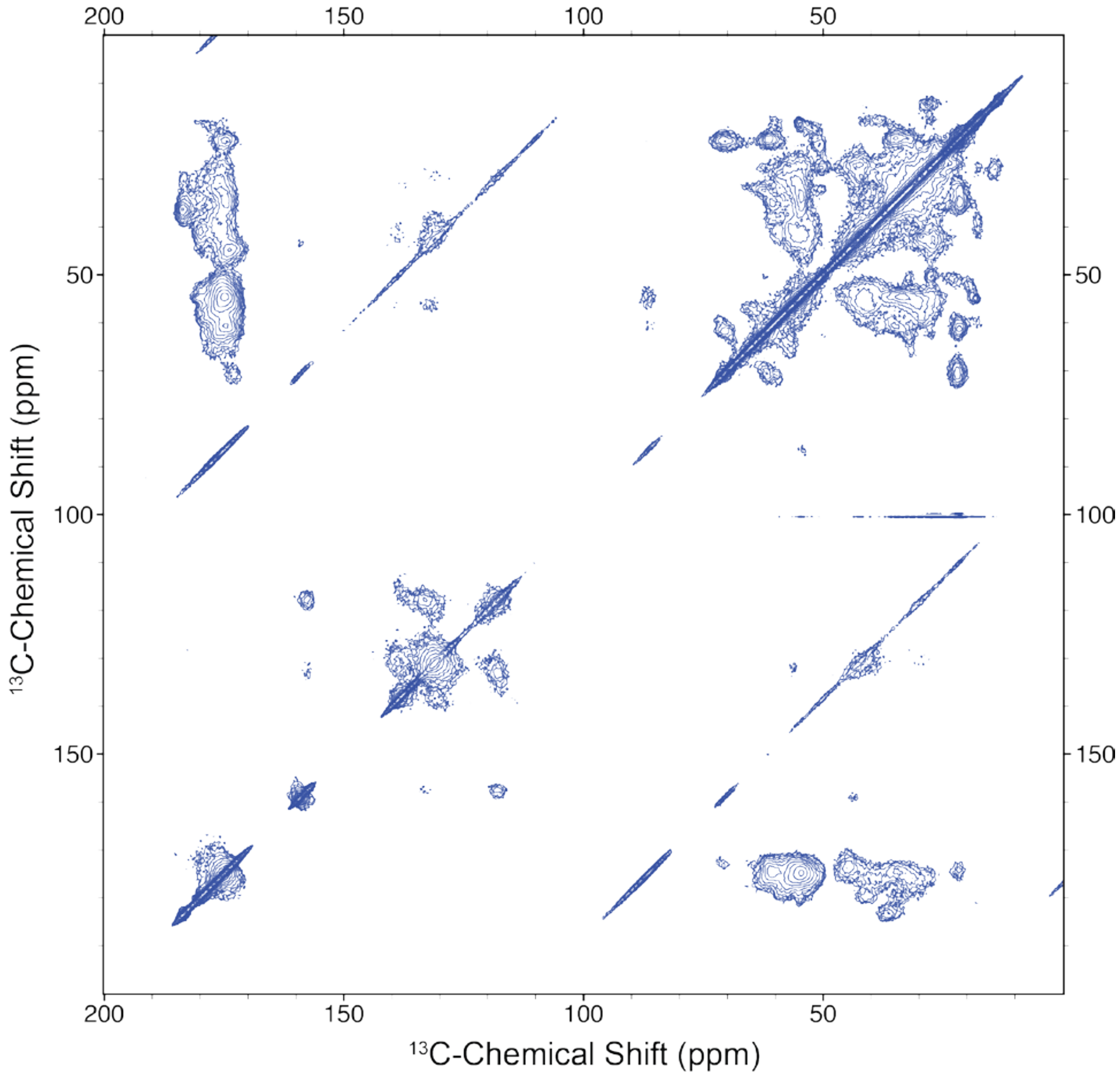

Figure S1. 2D ^{13}C-^{13}C DARR spectrum of CRES3. Full ^{13}C-^{13}C correlation spectrum of CRES3. Data was acquired with 12ms DARR mixing. A Lortenztizian-to-gaussian transformation was applied in each dimension with 50 Hz net Gaussian apodization.

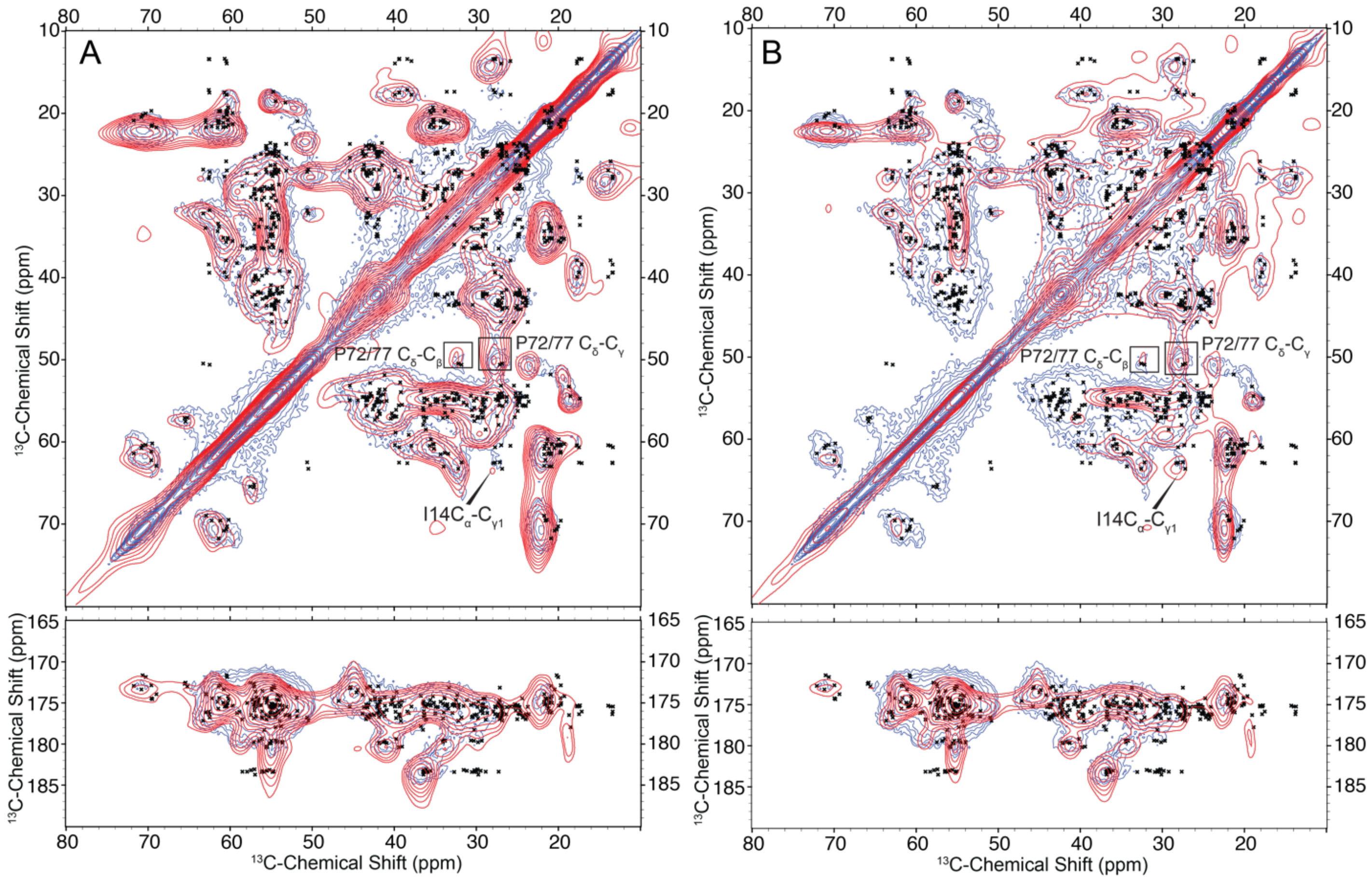

Figure S2. TIDE analysis of ^{13}C-^{13}C DARR spectrum of CRES3 amyloid. The TIDE deconvoluted spectrum is depicted for regions of the spectrum corresponding to aliphatic-aliphatic (top) and carbonyl-aliphatic (bottom) correlations. **A)** The TIDE processed spectra (red) constituting the last 60% of the T_2* envelope is overlaid onto the conventionally Fourier transformed spectrum (blue). This plane of the pseudo-3D TIDE spectrum contains resonances with shorter T_2*s, which should occupy more rigid regions of the CRES3 amyloid. **B)** The TIDE processed spectra (red) constituting the last 20% of the T_2* envelope is now overlaid onto the same conventionally processed Fourier transformed spectrum (blue). This plane is dominated by resonances with longer T_2*s, which should populate more dynamic regions of the protein. In both tiles, black crosses denote predicted chemical shifts from a domain-swapped model shown in Fig. 3C. Predicted chemical shifts discussed in the main text are specifically marked in the spectra. We can observe predicted resonances near the N-terminus (I14) are more dynamic compared to residues near the purported CRES loop (P72 and P77), which are indicative of a more well-ordered β-sheet structure. In addition to preliminary approximation of the dynamic properties of amyloidogenic CRES3, the TIDE deconvolution confirms the agreement between the observed chemical shifts and those predicted from the structural model are not an artifact of broad line widths and spectral crowding, but are largely representative of CRES3 secondary structure within the amyloid matrix.

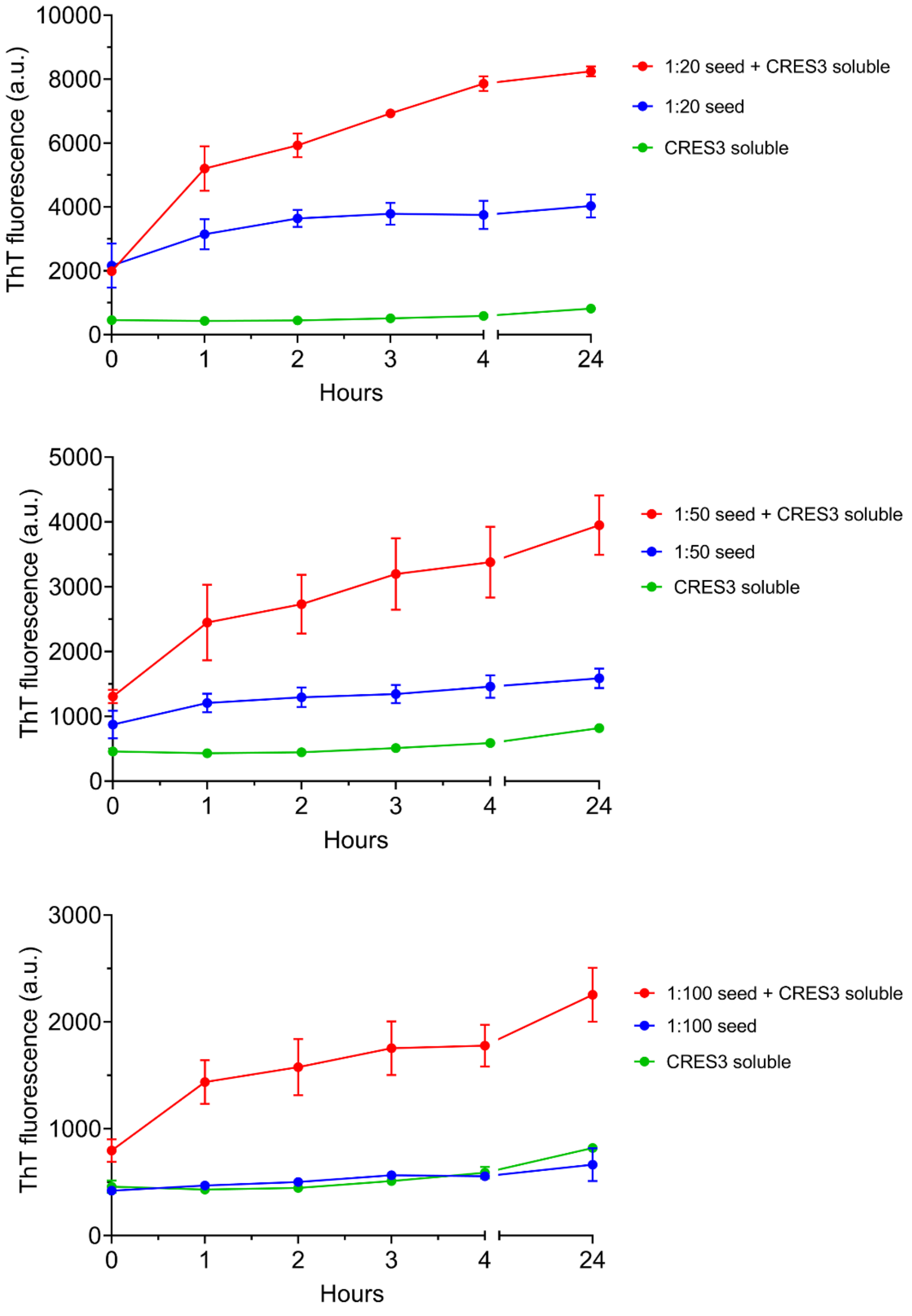

Figure S3. Self-seeding of CRES3 soluble fraction with different dilutions of CRES3 beaded chain seeds. Five µl of increasing dilutions of CRES3 seeds (1:20, 1:50, 1:100) were added to 10 µM CRES3 soluble fraction and assembly of amyloid followed over time by ThT plate assay. Data shown are the mean ±SEM of 3 independent experiments using 3 different CRES3 protein preparations. The error bars for the CRES3 soluble fraction are too small to be detected.

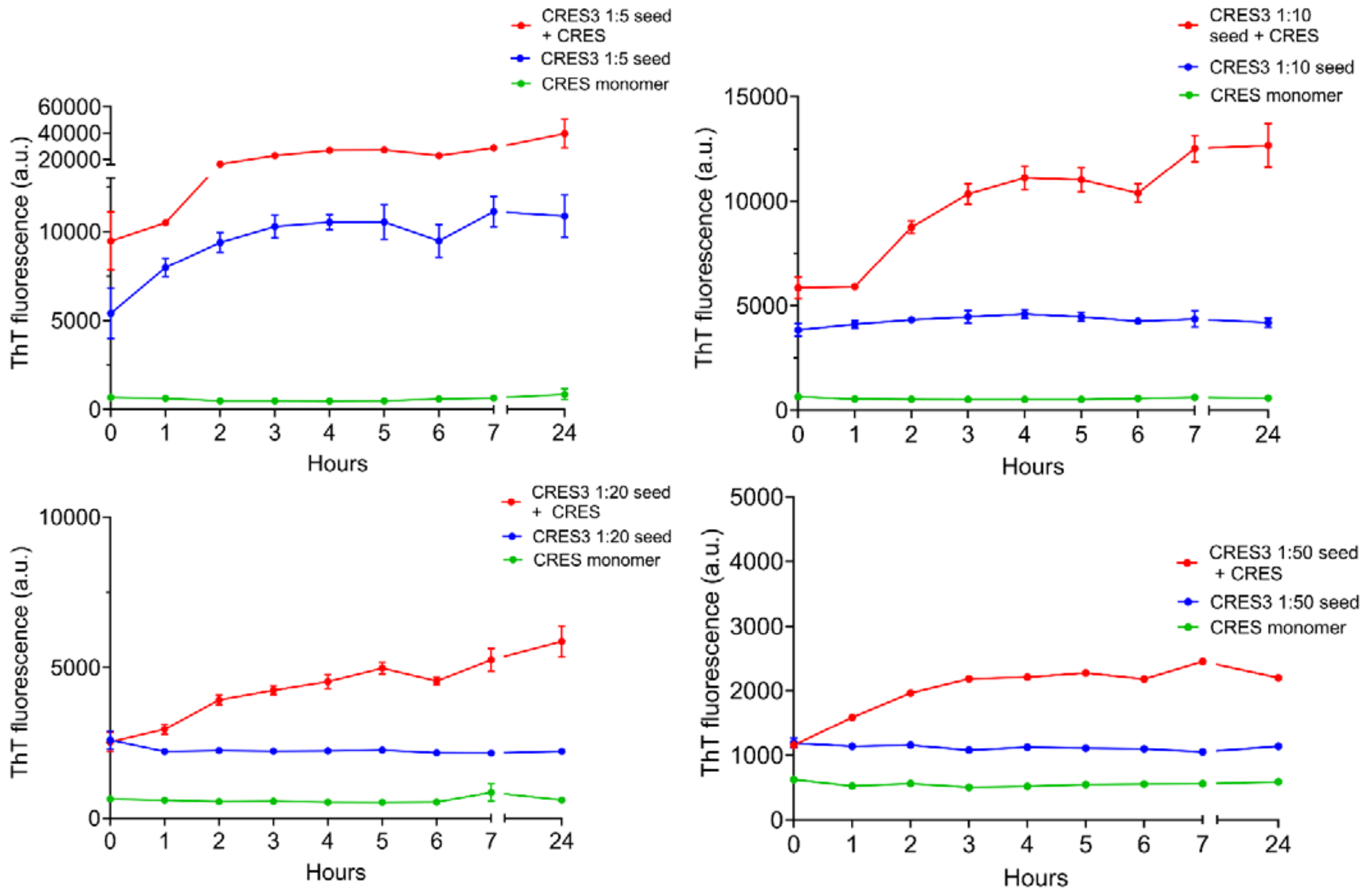

Figure S4. CRES3 exhibits dose-dependent cross-seeding of CRES monomer. Different dilutions of CRES3 seed (1:5, 1:10, 1:20, 1:50) were incubated with 10 μM CRES monomer and amyloid assembly followed by ThT plate assay. Data show the mean ± SEM of 6-9 replicates from 3 independent experiments. Error bars for CRES monomer and CRES3 seeds at some time points are too small to be detected.

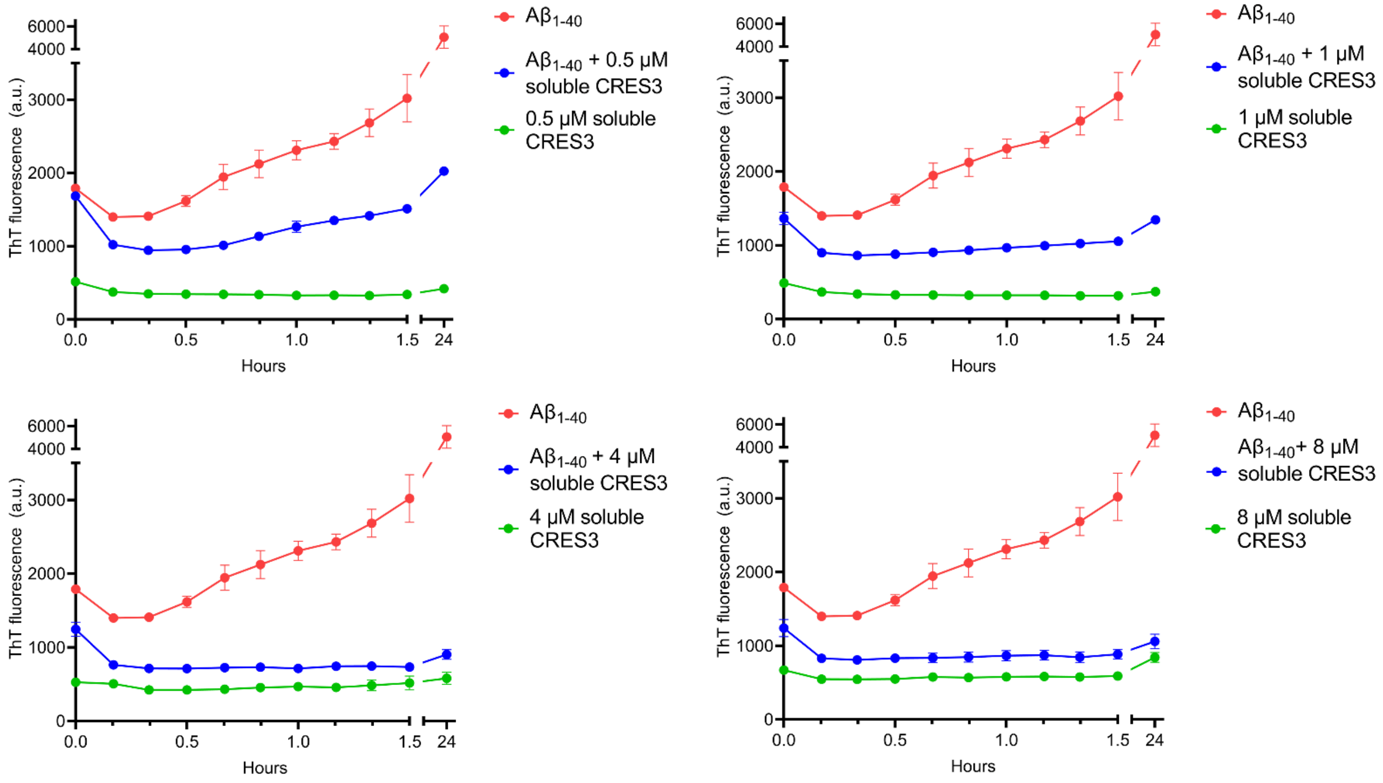

Figure S5. Soluble CRES3 inhibits Aβ$_{1\text{-}40}$ amyloid assembly in a dose-dependent manner. 0.5-8 μM soluble CRES3 was incubated with 8 μM Aβ$_{1\text{-}40}$ in a ThT plate assay. Amyloidogensis was followed for 1.5 hrs at 37°C after which the plates were sealed to prevent evaporation and then read again after 24 hours. Data represent the mean ±SEM of 9-12 replicates from 3 different experiments. Error bars for soluble CRES3 alone and Aβ/CRES3 are too small to be detected. The Aβ alone sample is the same in each graph since all dilutions were run in the same assay. The data are presented separately for clarity.

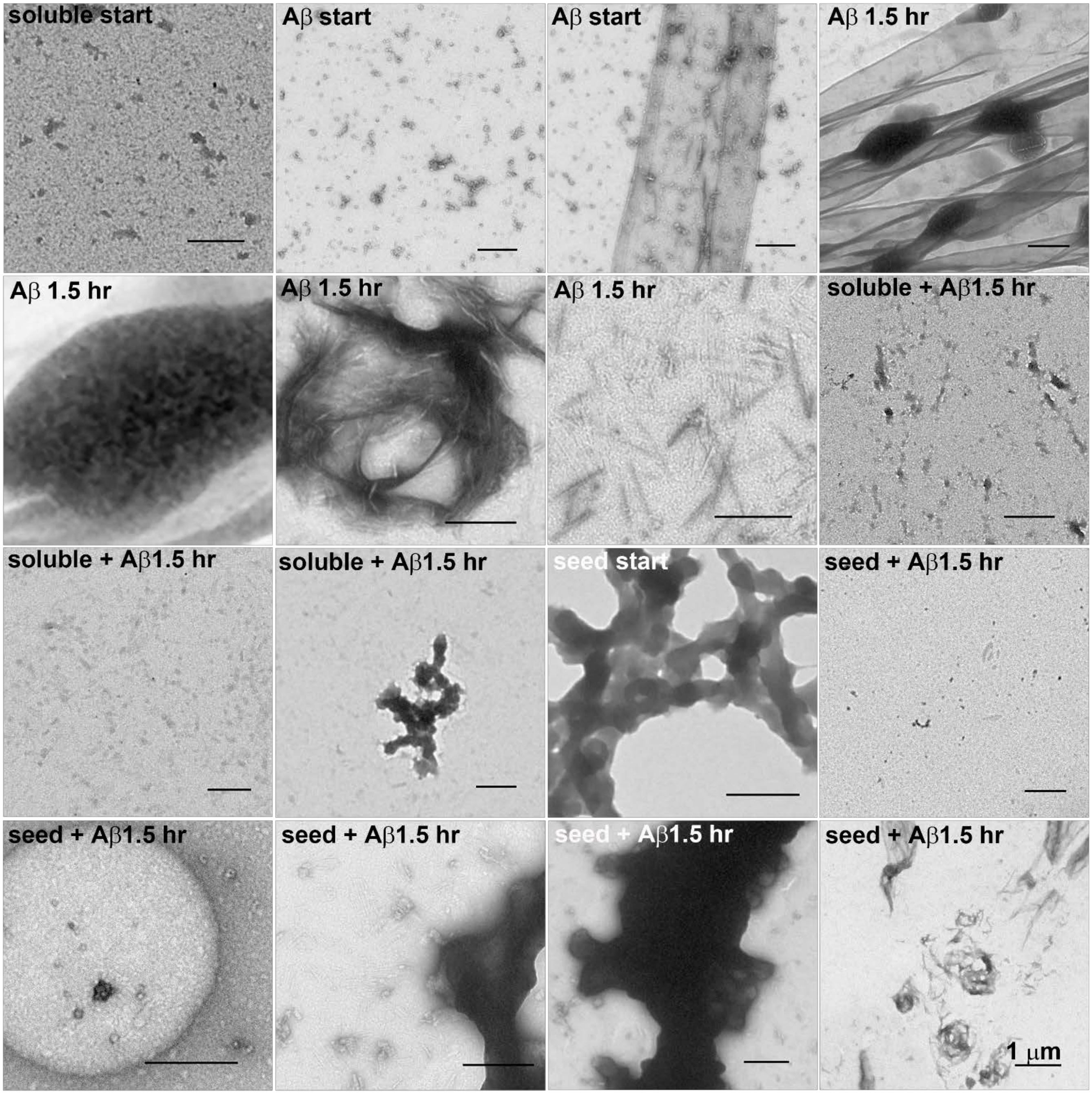

Figure S6.TEM of CRES3 and Aβ seeding reactions after 1.5 hours. ThT reactions from Fig 7A,B were spotted on to grids, stained with uranyl acetate and examined by TEM. Soluble CRES3 alone (8 μM) at time 0 (soluble start); CRES3 seed alone (1:20) at time 0 (seed start); Aβ alone at time 0 (start) and after 1.5 hours, and 8 μM soluble CRES3/Aβ and CRES3 seed (1:20)/Aβ coincubations after 1.5 hours are shown. Scale bar, 250 nm unless indicated.

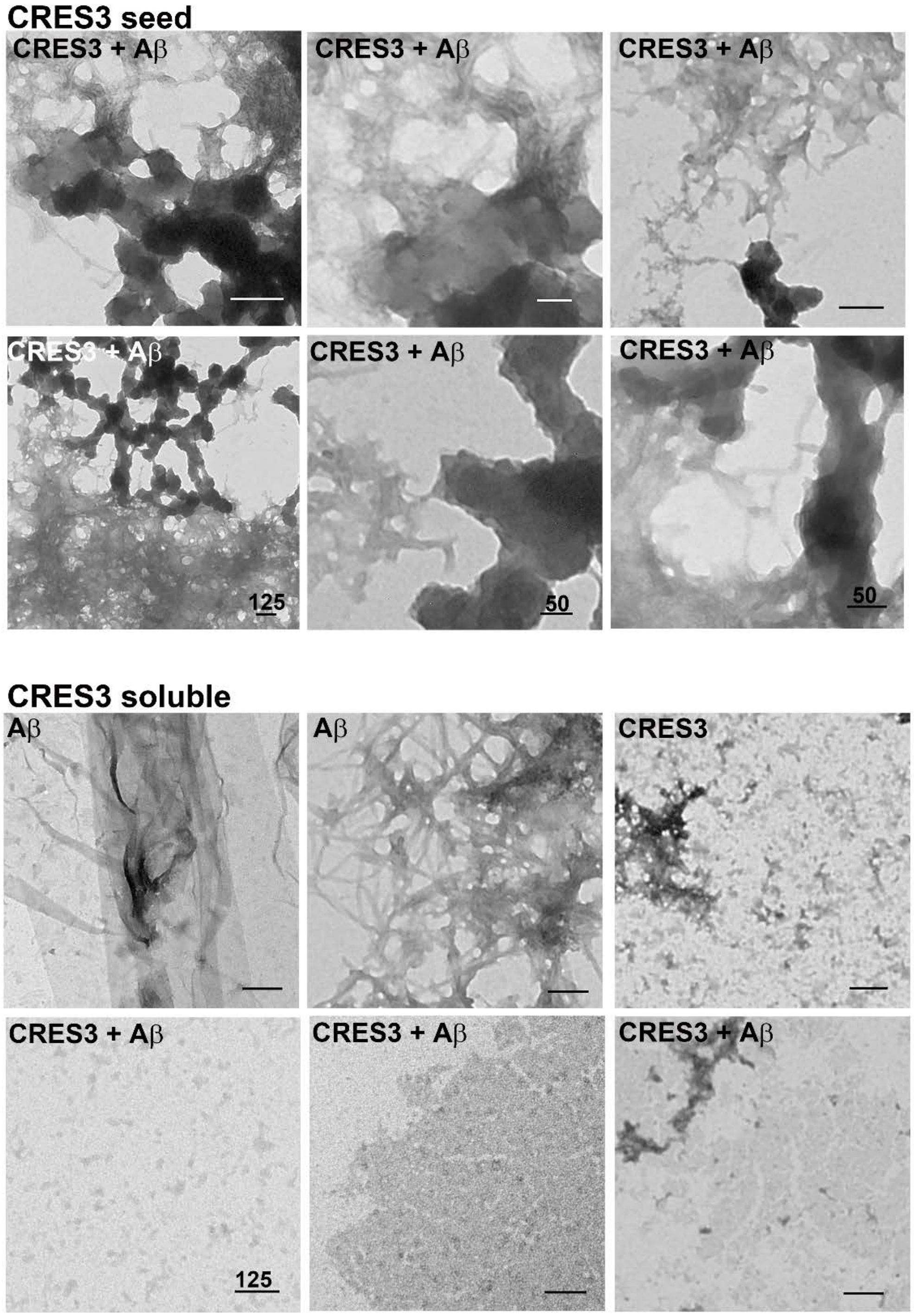

Figure S7. TEM of CRES3 and Aβ seeding reactions after 24 hours. ThT reactions from Fig 7A,B were spotted on to grids, stained with uranyl acetate and examined by TEM. CRES3 seed (1:20)/Aβ samples, 8 μM soluble CRES3 alone, Aβ alone, and 8 μM soluble CRES3/Aβ samples after 24 hours are shown. Scale bar, 250 nm unless indicated otherwise.

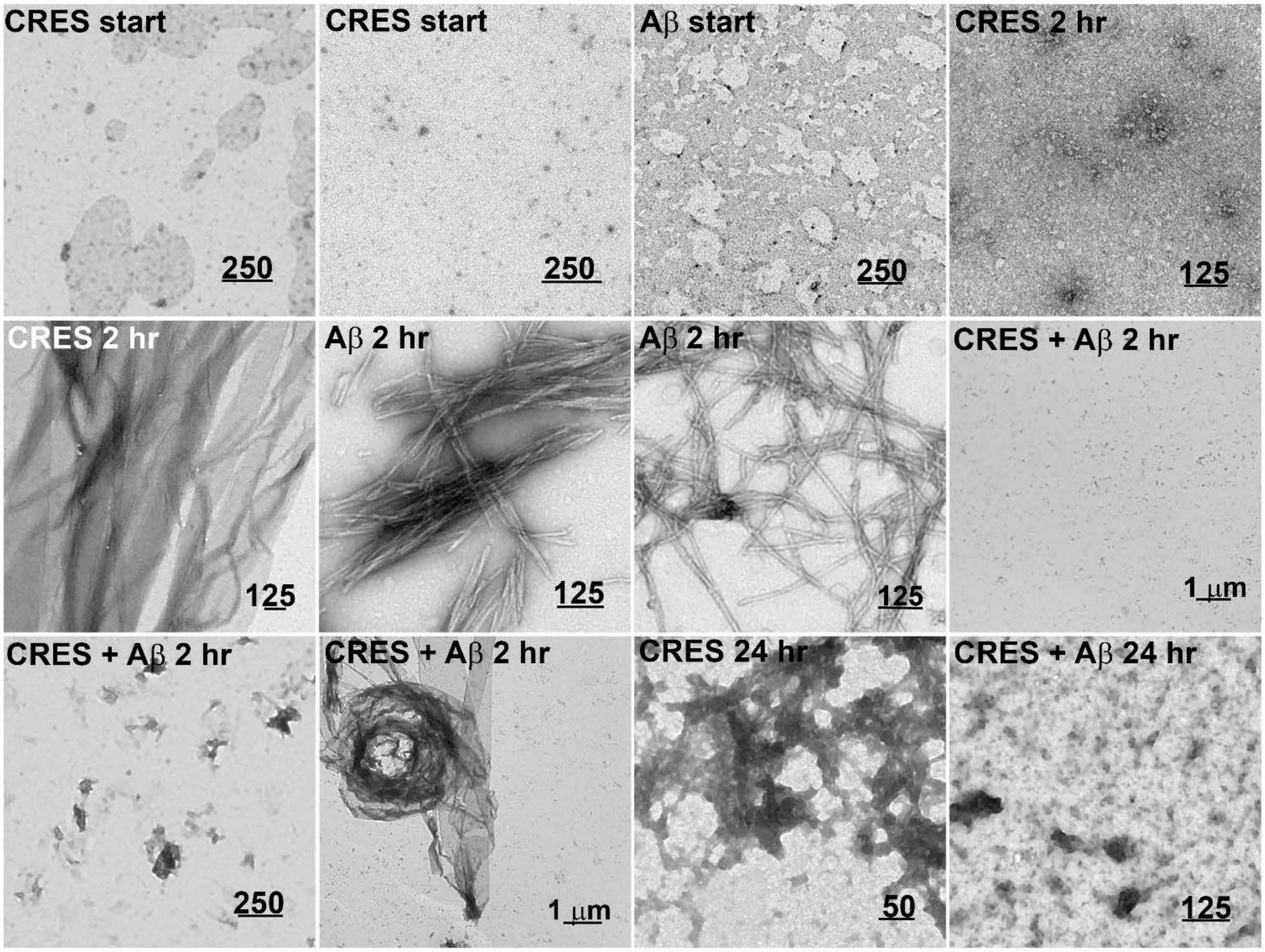

Figure S8. TEM of CRES C48A and Aβ seeding interactions after 2 and 24 hours. ThT reactions from Fig 7C were spotted on to grids, stained with uranyl acetate and examined by TEM. CRES C48A monomer alone at time 0 (start), 2 hours, and 24 hours, Aβ alone at time 0 (start) and 2 hours, and CRES C48A monomer/Aβ coincubations after 2 and 24 hours are shown. Scale bar, nm unless indicated otherwise.
